# Supplementary material for: RNA Promotes Synapsin Condensates That Organize Synaptic Vesicles in Live Neurons and Enable Localized Translation in Reconstituted System
Source: Adv Sci (Weinh). 2026 Aug 21;13(60):e77115. doi: 10.1002/advs.77115 (PMC13496291; doi:10.1002/advs.77115)
Supplement: Supplementary file 1 [file ADVS-13-e77115-s001.docx]

**Supplementary Data File for:**

**RNA promotes synapsin condensates that organize synaptic vesicles in live neurons and enable localized translation in reconstituted system**

Branislava Rankovic^1,2,3#^, Zachary M Geisterfer^1,4,#^, Akshita Chhabra^1,2,§^ , Vladimir M Jovanovic^5,§^, Ian Seim^3,11§^, Christian Hoffmann^1,2,§^, Antonela Condric^2^, Gerard Aguilar Pérez^1,2^, Rinse de Boer^7^, Lars T. Hofstede^7^, Jingao Sun^2^, Kiara Freitag^8^, Katja Nowick^5^, Marina Jendrach^8^, Frank L. Heppner^8^, Vladimir Despic^9^, Michaela Müller-McNicoll^9^, Stephan Sigrist^3^, Geert van den Bogaart^7^, Sofiia Reshetniak^10^, Amy S. Gladfelter^2,3,*^, Dragomir Milovanovic^1,2,11,*^

1 Whitman Center, Marine Biological Laboratory, 02543 Woods Hole, MA, USA

2 Institute of Biochemistry, Charité-Universitätsmedizin Berlin, Corporate Member of Freie Universität Berlin, Humboldt-Universität Berlin, and Berlin Institute of Health & Laboratory of Molecular Neuroscience, German Center for Neurodegenerative Diseases (DZNE), 10117 Berlin, Germany

3 Institute of Biology, Freie Universität Berlin, 14195 Berlin, Germany

4 Department of Cell Biology, Duke University, Durham, North Carolina 27708, USA

5 Human Biology and Primate Evolution, Freie Universität Berlin, 14195 Berlin, Germany

6 Present address: Max Planck Institute for Molecular Cell Biology and Genetics, 01307 Dresden, Germany

7 Department of Molecular Immunology, Groningen Biomolecular Sciences and Biotechnology Institute, University of Groningen, 9147 Groningen, The Netherlands

8 Department of Neuropathology, Charité - Universitätsmedizin Berlin, corporate member of Freie Universität Berlin, Humboldt-Universität zu Berlin and Berlin Institute of Health, 10117 Berlin, Germany.

9 Goethe University Frankfurt, Institute for Molecular Biosciences, Max-von-Laue-Str. 13, 60438 Frankfurt am Main, Germany

10 Institute for Neuro- and Sensory Physiology, University Medical Center Göttingen, 37073, Göttingen, Germany

11 Einstein Center for Neuroscience, Charité-Universitätsmedizin Berlin, Corporate Member of Freie Universität Berlin, Humboldt-Universität Berlin, and Berlin Institute of Health, 10117 Berlin, Germany

\# These authors contributed equally.

§ These authors contributed equally.

* Correspondence to: amy.gladfelter@duke.edu; dragomir.milovanovic@charite.de

**Supplementary Data File includes:**

- Supplementary Figures S1-S16;
- Supplementary Tables S1-S5.

**Supplementary Data Figures and Figure legends**

**Supplementary Figure 1| Phase diagram of RNA-driven synapsin-1 condensates.**

**(A)** Phase separation of purified recombinant EGFP-synapsin-1 full-length (Syn 1) protein was assessed with respect of increasing concentrations of protein and total neuronal RNA. *In vitro* reconstitution of 10 µl-reaction mix of Syn 1 and RNA was prepared under physiological salt conditions and in the absence of any molecular crowders (150 mM NaCl, 25 mM Tris-HCl pH 7.4 and 0.5 mM TCEP). These representative images correspond to the phase diagram plot in **Fig. 1B**.

(**B)** Phase diagram as a function of total charge of synapsin-1, calculated as number of synapsin-1 moles in the reaction mixture multiplied with z (+20.523) for Syn 1, and RNA, calculated as a charge of the total neuronal RNA phosphate backbone charge. The colored histogram depicts the ratio of charges in Syn 1 and RNA.

**Supplementary Figure 2 | Synapsin-1/RNA condensates recruit synaptic vesicles *in vitro.***

**(A)** Representative micrographs of *in vitro* reconstitutions of 10 µM EGFP-synapsin-1 (Syn 1, in green) with 300 ng total neuronal RNA condensates with (+) and without (-) SV addition. To visualize SVs membrane staining FM4-64 styryl dye was used (in magenta).

**(B)** Quantification of the diameter of Syn 1/RNA condensates with (+) and without (-) SV addition. The plot represents frequency distribution of Syn 1 condensates diameter with (+) or without (-) SVs.

**(C)** FRAP curves of Syn 1/RNA condensates (+) or (-) SVs. Fluorescent recovery after photobleaching was recorded for 600 s with an interval of 1 s. No significant effect was observed on the Syn 1/RNA condensates dynamics upon addition of SVs. Fractional recovery ±SD was plotted as a function of time. Data were collected from three independent FRAP experiments.

**Supplementary Figure 3 | RNase A treatment of synapsin-1/RNA condensates.**

**(A)** Schematic representation of *in vitro* RNase A assay workflow.

**(B)** Quantification of EGFP-synapsin-1 (Syn 1) Syn 1 fluorescence enrichment in Syn 1/RNA condensates (10 µM Syn 1 to a final concentration and 300 ng of total neuronal RNA) just before and upon RNase A addition (to a final concentration of 0.1-green, and 1 µg/µl-magenta) was assessed in the course of 10 min incubation time. Partition coefficient (±SD) of Syn 1 for 10 manually selected condensates per technical replicate for each condition was analyzed. Green dashed line indicates first value of fluorescence intensities of Syn 1 condensates quantified immediately upon RNase A addition. Data were collected from three independent *in vitro* reconstitutions (total 30 selected condensates). This quantification corelates with representative images of selected condensate in **Fig. 1D**.

**(C)** Quantification of Syn 1 fluorescence intensity of a dilute phase before and 10 min after the treatment with 1 (left) or 0.1 µg/µl (right) of RNase A (complementary to data in **Fig. 1E**). The jitter box plot encompasses 25th and 75th quartiles with median marked by central line. Minimum and maximum of quantified Syn 1 fluorescence intensities are denoted by whiskers. Black circles (above the whiskers) represent quantified fluorescence intensities outside the range of adjacent values. Each point represents the value, which was slightly shifted for better visualization of overlapping data points. **** indicates P ≤ 0.0001, *** P ≤ 0.001 by Wilcoxon signed-rank test. Data was collected from three independent in vitro reconstitutions.

**(D)** Representative micrographs indicating that 10 µM Syn 1 readily forms condensates with intact RNA (left) but fails to phase separate in the presence of nucleotides (i.e., predigested RNA with 1 µg/µl of RNase A; right).

**(E)** Coomassie gel of Syn 1 and RNase A used in the assay. Note that Syn 1 remains intact in the presence of RNase A. The original image of the gel is shown in the Supporting Information file.

**(F)** Representative micrographs of Syn 1 condensates (10 µM) with RNA (20 ng/µL) treated with 1 µg/µL RNase A of regular (left) and reduced (right) activity. Reduced activity RNase A (red. activity) was boiled for 45 min at 98 °C in the presence of 20 mM DTT and supplemented with RNaseOUT (0.25 U/µg of RNase A) upon cooling.

**(G)** Line profile of regions highlighted in F for regular RNase A (magenta) and reduced activity RNase A (blue) before and after addition of RNase A. Note that red. activity RNase A led to a substantially less dispersion of synapsin-1. Results were reproduced in two independent reconstitution experiments.

**Supplementary Figure 4 | Syn 1-IDR can recruit more unstructured mRNAs *in vitro*.**

**(A)** Representative images of *in vitro* reconstituted EGFP-synapsin-1-IDR (Syn 1-IDR) condensates (green) enriched with Cy5-labelled high-ED *CLN3* mRNA (yellow) of *A. gossypii*.

**(B)** Plot showing fluorescence intensities quantification of colocalizing Syn 1-IDR and *CLN3* high-ED in corresponding Syn 1-IDR/*CLN3* high-ED condensates. In the scatter plot overlayed with Kernel Density Estimate (KDE) each circle represents Syn 1-IDR/RNA condensate analyzed. Data were collected from three independent *in vitro* reconstitutions.

**Supplementary Figure 5 | In vitro reconstitution of synapsin-1 and α-synuclein with *CLN3* high ED mRNA.**

Representative micrographs of *in vitro* reconstituted synapsin-1/α-synuclein (Syn 1/αSyn) condensates with or without *CLN3* high ED mRNA upon 15 min of incubation under physiological salts conditions and in the absence of molecular crowders.

**Supplementary Figure 6 | SV-like condensates purified from HEK293 cells contain RNA with motifs of higher GC content and a lower total normalized ensemble diversity.**

**(A)** Representative micrographs of HEK293 cells co-transfected with mCherry-synapsin-1 and untagged synaptophysin (green, condition 1 [Syn 1 condensates]) and mCherry-synapsin-1 together with untagged α-synuclein and synaptophysin (magenta, condition 2 [Syn1/αSyn condensates]). Synaptophysin was used to promote synapsin-1 condensation *in cellulo*. Third image in the row: HEK293 cells expressing only mCherry tag used to set up the gate parameters for fluorescence activated sorting of purified synapsin-1 or synapsin-1/α-synuclein condensates.

**(B)** Schematic representation of condensate purification protocol adapted from [Hubstenberger et al., 2017].

**(C)** representative images of lysate and pellet granule fraction along the purification process of mCherry-Syn 1 (condition 1, Syn 1) and mCherry-Syn 1+αSyn (condition 2, Syn 1/αSyn) condensates. Selected images show overlay of transmitted light (gray) and mCherry fluorescence (black). Black arrows indicate condensate of interest. Orange asterisks indicate membrane debris and magenta asterisks indicate other granules of 10 000× g fraction. Fractions were collected through the process of SV-like condensate purification and immediately imaged.

**(D)** Western blot of mCherry-Syn 1 from two different conditions (1: Syn 1 and 2: Syn 1/αSyn, expressing HEK293 cells). Recombinant mCherry-Syn 1 was used as a positive control (app 0.8 µg was loaded on the gel). β-Actin was used as loading control. For each fraction approx. 12 µg of total protein was loaded on the gel. Anti-mCherry and anti- β-actin were used to detect proteins of interest. Contrast and brightness were adjusted for optimal visualization. The original and uncropped images of the blot is shown in the Supporting Information file.

**(E)** Example of mCherry particles flow analysis represented as forward scatter (FSC-A) as a function of mCherry fluorescence (PE-Texas-Red A-mCherry). Dark red selection line show subset value against mCherry dispersed signal later used as a gate for sorting of condensates of interest.

**(F)** Flow analysis of SV-like purified condensates represented as forward scatter (FSC-A) as a function of mCherry fluorescence (PE-Texas-Red A-mCherry). Dark red selection line show subset of sorted condensates. Plots show two biological replicates Syn 1 and Syn 1/αSyn condensates.

**(G)** Flow analysis of SV-like purified condensates represented as forward scatter (FSC-A) as a function of mCherry fluorescence (PE-Texas-Red A-mCherry). Only sorted fractions was presented in density plots of two biological replicates of Syn 1 and Syn 1/αSyn condensates.

**(H)** Sequence logos depicting short stretches of statistically overrepresented sequences found in the transcriptomes of ectopically expressed Syn1 (green) and Syn1/αSyn (magenta) condensates. The number of transcripts that contain these sequences within each transcriptome are listed above each sequence logo. To be considered significant, sequence motifs needed to have an E-value < $1\times{10}^{-3}$. E-values for sequence 1, 2, 3, and 4 are $7.7\times{10}^{-165}$, $1.3\times{10}^{-140}$, $1.3\times{10}^{-384}$, and $1.6\times{10}^{-88}$, respectively.

**(I)** Plot representing normalized ensemble diversity (NED) of transcript from designated RNAomes (x-axis) normalized by transcript length.

**Supplementary Figure 7 | ED signature of the synaptic RNAome data set.**

Ensemble diversity (ED) analysis plot showing synaptic transcripts from [57] S1 data set in correlation with *CLN3* low-ED and *CLN3* high-ED *Ashbya gossypii* mRNAs. *Prnp* and *Scg3* mRNAs (highlighted in the plot with a orange and grey star, respectively) were used in the *in vitro* reconstitution experiments of **Fig. 3**. The scatter plot shows transcript ED values as a function of transcript length. Each circle represents an individual mRNA. nt stands for nucleotides.

**Supplementary Figure 8 | Synaptic mRNAs affect α-syn recruitment into synapsin-1 condensates.**

**(A)** Representative scheme of type I *in vitro* reaction set up (see Methods): First protein-protein condensates of synapsin-1 (Syn 1) and α-synuclein **(**αSyn) were formed and loaded on glass bottom dish for subsequent imaging. After approx. 7 min mRNA of interest was added precisely into the reaction droplet. Next, for mRNA and protein enrichment assessment images were taken 10- and 25-min post proteins mixing step.

**(B)** Representative images of type I *in vitro* reaction set up at 10 min after synaptic mRNA (*Scg3* or *Prnp*) addition.

**(C)** Representative scheme of type II *in vitro* reaction set up (see Methods): First Syn 1 and mRNA of interest were pre-mixed in the reaction tube and incubated for 1 min. Next, αSyn was added and the reaction mix was aliquoted on the glass bottom dish for subsequent imaging. For mRNA and protein enrichment assessment images were taken 5- and 25-min post reaction set up.

**(D)** Representative images of type II *in vitro* reaction set up at min 5 after mixing all the reaction components.

**(E)** Quantifications of mRNA enrichment (*Scg3* or *Prnp*) in *in vitro* reconstituted RNA-protein droplets at time points t1 and t2 (reaction set up type II, see Methods). Box plots (e, f) encompass the 25^th^ and 75^th^ quartiles with the median marked by central line. Whiskers extend to 1.5 times the interquartile range (IQR). Circles indicate outliers. Paired two-tailed T-test comparing the intensities of RNA within condensates at t1 and t2 results in a P-value of <0.00001 for reactions containing either *Scg3* or *Prnp*. Data were collected from three independent *in vitro* reconstitutions.

**Supplementary Figure 9 | Synaptic mRNAs phase separates with synapsin-1 *in vitro*.**

**(A)** Representative images of *in vitro* reconstituted EGFP-synapsin-1 (Syn 1) condensates with Cy3-labelled synaptic mRNAs (*Prnp* or *Scg3*) after approx. 20 min of incubation upon reaction set up in the reaction buffer with physiological salt conditions and the absence of any molecular crowder (see Methods).

**(B)** Quantification of RNA fluorescence intensities (*Scg3* in grey and *Prnp* in magenta) as a function of Syn 1 fluorescence intensities. In the scatter plot overlayed with Kernel Density Estimate (KDE) each circle represents Syn 1/RNA condensate analyzed. Data were collected from three independent *in vitro* reconstitutions.

**Supplementary Figure 10 | Validation of PCI method in mammalian cells.**

**(A)** Schematic representation of validation of the PCI method in HEK293 cell line.

**(B)** Representative images of HEK293 cells at each step of PCI. Dextran-FITC (green) and dextran-AlexaFluor (AF)546 (magenta) were used to assess internalization of small molecules and their retention 30 min post photo-activation, as well as membrane sealing following photo-activation. Nuclei (blue) were stained with Hoechst 33342.

**Supplementary Figure 11| PCI-mediated RNase A treatment in primary rat neurons.**

**(A)** Representative micrographs of full frame axonal network before and after RNase A treatment and

**(B)** before and after BSA treatment showing mScarlet-synapsin (green) 1 and synaptophysin-EGFP (magenta) signal distribution at the synaptic boutons. These images correspond to the axonal segments in **Fig. 4***.*

**(C)** Representative micrographs of axonal segments before and after treatment by light exposure (left) or incubation with RNase A (right) in the same experimental conditions as in results presented in **Fig. 4**, but in the absence of the photosensitizer. Black circles indicate presence of the specific condition while white circles indicate the absence of the same.

**(D)** Bar-plot representing total count of analyzed boutons before and after RNase A (left) and BSA treatment (right). In total 61 synapses from 4 independent cultures were analyzed for RNase A treatment. 60 synapses from 3 independent cultures were analyzed for BSA treatment.

**(E)** Bar plot representing percentage of boutons that underwent dispersion event (defined as >30 % of mScarlet-synapsin-1 fluorescence signal decrease in a bouton) upon RNase A treatment compared to BSA control.

**(F)** Representative images of axonal segment before and after RNase A treatment followed by immunostaining of post synaptic marker PSD-95 (in blue). Synapsin 1 is in magenta, synaptophysin in green.

**Supplementary Figure 12 | Quality control of PCI mediated RNase A treatment in primary neurons.**

Representative images for quality control of RNase A treatment in primary rat neurons showing that poly(A) signal was RNase A-sensitive. Left: image shows poly(A) FISH signal of neurons treated with BSA for 30 min. Right: image shows primary neuron underwent RNase A treatment for 30 min to a final concentration of RNase A of 150 µg/ml. Orange asterisks indicate neuronal soma. Glial cells could be detected in the background with noticeable poly(A) signal depletion in treated example. Images of BSA and RNase A-treated neurons were acquired under the exact same imaging conditions.

**Supplementary Figure 13 | Synapsin-1 condensates harbor mRNAs and EIF4E.**

**(A)** Representative full frame image of hippocampal axon showing poly(A) FISH and vGLUT1 immunostaining. Selected region corresponds to the neurite segment of mouse primary neurons in **Fig. 5A**. Arrows indicate additional examples of poly(A) (magenta) co-localizing with vGLUT1 signal (green). Scale bar: 20 µm.

**(B)** Representative full frame image of hippocampal neurons showing *Atp5b* smFISH signal (magenta) and vGLUT1 immunostaining (green). MAP2, a neuron specific marker is in blue and DAPI in cyan. Selected region corresponds to the neurite segment in **Fig. 5C**. Scale bar: 20 µm.

**(C)** Representative images of neurite segment selected in **(B)** indicating detected *Atp5b* foci (using radial symmetry-FISH; white circles overlay) from **Fig. 5C** and corresponding raw smFISH signal of *Atp5b* in magenta (image below). vGLUT1 is indicated in green (last image of panel C).

**(D)** Schematic representation of *Atp5b* transcript (*Mus musculus*, NM_016774.3) and 30 selected 18-20mer sequences used to design smFISH probes (light green) spanning over the *Atp5b* CDS and 3’UTR.

**(E)** Validation of anti-EIF4E antibody (abcam; ab33766) in not treated and 30 min Arsenite (330 µM) treated HEK293 cells. As expected EIF4E re-localize in stress granules also positive for poly(A) enrichment (magenta). DAPI is in cyan. Scale bar: 10 µm and 3 µm in selected inset. Arrows in selected inset indicate EIF4E (yellow; immunostained with anti-EIF4E) and poly(A) (magenta; FISH signal) positive stress granule.

**(F)** Representative full frame image of HEK293 cells expressing EGFP-synapsin-1 (Syn 1, green) and synaptophysin (untagged) stained with anti-EIF4E antibody (magenta). Scale bar, 10 µm.

**(G)** Representative full frame image of HEK293 cells expressing EGFP-tagged SH3(AB) concatemer of intersectin-1 (ITSN1-AB, green) stained with anti-EIF4E antibody (magenta). Scale bar, 10 µm.

**(H)** Magnified inset from F, region highlighted with dotted line. Scale bar, 5 µm.

**(I)** Line profile across EGFP-synapsin-1 condensates from H.

**(J)** Magnified inset from G, region highlighted with dotted line. Scale bar, 3 µm

**(K)** Line profile across ITSN1-AB condensates from J. Note the absence of anti-EIF4E antibody signal.

**(L)** Pie-chart of (top): quantified Syn 1 condensates (n_condensate_=209) containing EIF4E positive signal; (bottom): quantified ITSN1-AB condensates (n_condensate_=165) containing EIF4E positive puncta. Syn 1 condensates of diameter 1-4 µm were analyzed. Same size cut-off was applied for ITSN1-AB condensates. Syn 1 condensates were analyzed in 115 cells and ITSN1-AB condensates in 98 cells (indicated with n_cells_). Data was collected from three independent biological replicates for both conditions (indicated with N).

**(M)** Graph showing fraction of synapsin 1 condensates containing EIF4E in comparison to ITSN1-AB condensates.***, P-value < 0.001 by two-tailed, unpaired Student’s t-test.

**Supplementary Figure 14 | CLEM data of ribosomes localized in the vicinity of synapsin-1 condensates.**

**(A)** Fluorescence microscopy image of a 200 nm section of cryo-fixed HEK293 cells expressing EGFP-synapsin-1 and synaptophysin, overlaid with a TEM image of the same region. Scale bar, 2 µm.

**(B)** Tomographic reconstruction and 3D-rendered volume of the region indicated by the dashed box in *A*. Vesicular condensates are shown in cyan and ribosomes in orange. Scale bar, 200 nm.

**Supplementary Figure 15 | MoonTag in vitro system: noRNA control reaction.**

Representative images of MoonTag in vitro reaction upon addition of rabbit reticulocyte extract (RRE) to EGFP-synapsin-1 (top) and pre-formed *Atp5b::Moon*/EGFP-synapsin-1 condensates. Scale bars, 5 μm. Note that no Syn 1 condensation is promoted in the absence of *Atp5b::Moon* mRNA in the RRE.

**Supplementary Figure 16 | RNA driven synapsin-1 condensates are permissive for micro to mesoscale molecules.**

**(A)** Representative scheme of *in vitro* reconstitution with Syn 1 (green), PEG 8000 and TMR-dextran (magenta) of specific molecular weight.

**(B)** Representative images of dextran assay for assessing the pore size of 4.5 µM Syn 1 condensates in the presence of 3 % molecular crowder PEG 8000 in the reaction buffer (150 mM NaCl, 25 mM Tris-HCl pH 7.4 and 0.5 mM TCEP).

**(C)** Representative scheme of *in vitro* reconstitution with Syn 1 (green), total neuronal RNA and TMR-dextran (magenta) of specific molecular weight.

**(D)** Representative images of dextran assay for assessing the pore size of 4.5 µM Syn 1 condensates in the presence of 150 ng of total neuronal RNA in reaction buffer (150 mM NaCl, 25 mM Tris-HCl pH 7.4 and 0.5 mM TCEP).

**(E)** Quantification of TMR dextran (sizes: 4.4, 65.85 and 155 kDa) enrichment in PEG- (left-hand panel plots) or RNA-driven (right-hand panel plots) Syn 1 condensates. Each plot represents line profiles of normalized fluorescence intensity signals with ±SEM of EGFP-Syn 1 (green) and TMR-dextran of different sizes (magenta). Data were collected from 3 independent *in vitro* reconstitutions.

**Supplementary Table S1: Plasmids used in this study.**

Table S1. Plasmids in use | insert sequence | IVT templates

Plasmids in use: | insert sequence | expression system | source

mCherry-hsSYNAPSIN 1 | mammalian | Hoffman, Sansevrino et al., 2021

ATGGGCCATCACCATCACCATCACAGCGGCAGCGAGAACCTGTACTTCCAGGGCGTGAGCAAGGGCGAGGAGGATAACATGGCCATCATCAAGGAGTTCATGCGCTTCAAGGTGCACATGGAGGGCTCCGTGAACGGCCACGAGTTCGAGATCGAGGGCGAGGGCGAGGGCCGCCCCTACGAGGGCACCCAGACCGCCAAGCTGAAGGTGACCAAGGGTGGCCCCCTGCCCTTCGCCTGGGACATCCTGTCCCCTCAGTTCATGTACGGCTCCAAGGCCTACGTGAAGCACCCCGCCGACATCCCCGACTACTTGAAGCTGTCCTTCCCCGAGGGCTTCAAGTGGGAGCGCGTGATGAACTTCGAGGACGGCGGCGTGGTGACCGTGACCCAGGACTCCTCCCTGCAGGACGGCGAGTTCATCTACAAGGTGAAGCTGCGCGGCACCAACTTCCCCTCCGACGGCCCCGTAATGCAGAAGAAGACCATGGGCTGGGAGGCCTCCTCCGAGCGGATGTACCCCGAGGACGGCGCCCTGAAGGGCGAGATCAAGCAGAGGCTGAAGCTGAAGGACGGCGGCCACTACGACGCTGAGGTCAAGACCACCTACAAGGCCAAGAAGCCCGTGCAGCTGCCCGGCGCCTACAACGTCAACATCAAGTTGGACATCACCTCCCACAACGAGGACTACACCATCGTGGAACAGTACGAACGCGCCGAGGGCCGCCACTCCACCGGCGGCATGGACGAGCTGTACAAGTCCGGACTCGGCGGCCACCGCTGGATCCTGGAGGTGCTGTTCCAGGGCCCCAGATCTATGAACTACCTGCGCCGCCGCCTGAGCGACAGCAACTTCATGGCCAACCTGCCCAACGGCTACATGACCGACCTGCAGCGCCCCCAGCCCCCCCCCCCCCCCCCCGGCGCCCACAGCCCCGGCGCCACCCCCGGCCCCGGCACCGCCACCGCCGAGCGCAGCAGCGGCGTGGCCCCCGCCGCCAGCCCCGCCGCCCCCAGCCCCGGCAGCAGCGGCGGCGGCGGCTTCTTCAGCAGCCTGAGCAACGCCGTGAAGCAGACCACCGCCGCCGCCGCCGCCACCTTCAGCGAGCAGGTGGGCGGCGGCAGCGGCGGCGCCGGCCGCGGCGGCGCCGCCAGCCGCGTGCTGCTGGTGATCGACGAGCCCCACACCGACTGGGCCAAGTACTTCAAGGGCAAGAAGATCCACGGCGAGATCGACATCAAGGTGGAGCAGGCCGAGTTCAGCGACCTGAACCTGGTGGCCCACGCCAACGGCGGCTTCAGCGTGGACATGGAGGTGCTGCGCAACGGCGTGAAGGTGGTGCGCAGCCTGAAGCCCGACTTCGTGCTGATCCGCCAGCACGCCTTCAGCATGGCCCGCAACGGCGACTACCGCAGCCTGGTGATCGGCCTGCAGTACGCCGGCATCCCCAGCGTGAACAGCCTGCACAGCGTGTACAACTTCTGCGACAAGCCCTGGGTGTTCGCCCAGATGGTGCGCCTGCACAAGAAGCTGGGCACCGAGGAGTTCCCCCTGATCGACCAGACCTTCTACCCCAACCACAAGGAGATGCTGAGCAGCACCACCTACCCCGTGGTGGTGAAGATGGGCCACGCCCACAGCGGCATGGGCAAGGTGAAGGTGGACAACCAGCACGACTTCCAGGACATCGCCAGCGTGGTGGCCCTGACCAAGACCTACGCCACCGCCGAGCCCTTCATCGACGCCAAGTACGACGTGCGCGTGCAGAAGATCGGCCAGAACTACAAGGCCTACATGCGCACCAGCGTGAGCGGCAACTGGAAGACCAACACCGGCAGCGCCATGCTGGAGCAGATCGCCATGAGCGACCGCTACAAGCTGTGGGTGGACACCTGCAGCGAGATATTCGGCGGCCTGGACATCTGCGCCGTGGAGGCCCTGCACGGCAAGGACGGCCGCGACCACATCATCGAGGTGGTGGGCAGCAGCATGCCCCTGATCGGCGACCACCAGGACGAGGACAAGCAGCTGATCGTGGAGCTGGTGGTGAACAAGATGGCCCAGGCCCTGCCCCGCCAGCGCCAGCGCGACGCCAGCCCCGGCCGCGGCAGCCACGGCCAGACCCCCAGCCCCGGCGCCCTGCCCCTGGGCCGCCAGACCAGCCAGCAGCCCGCCGGCCCCCCCGCCCAGCAGCGCCCCCCCCCCCAGGGCGGCCCCCCCCAGCCCGGCCCCGGCCCCCAGCGCCAGGGCCCCCCCCTGCAGCAGCGCCCCCCCCCCCAGGGCCAGCAGCACCTGAGCGGCCTGGGCCCCCCCGCCGGCAGCCCCCTGCCCCAGCGCCTGCCCAGCCCCACCAGCGCCCCCCAGCAGCCCGCCAGCCAGGCCGCCCCCCCCACCCAGGGCCAGGGCCGCCAGAGCCGCCCCGTGGCCGGCGGCCCCGGCGCCCCCCCCGCCGCCCGCCCCCCCGCCAGCCCCAGCCCCCAGCGCCAGGCCGGCCCCCCCCAGGCCACCCGCCAGACCAGCGTGAGCGGCCCCGCCCCCCCCAAGGCCAGCGGCGCCCCCCCCGGCGGCCAGCAGCGCCAGGGCCCCCCCCAGAAGCCCCCCGGCCCCGCCGGCCCCACCCGCCAGGCCAGCCAGGCCGGCCCCGTGCCCCGCACCGGCCCCCCCACCACCCAGCAGCCCCGCCCCAGCGGCCCCGGCCCCGCCGGCCGCCCCAAGCCCCAGCTGGCCCAGAAGCCCAGCCAGGACGTGCCCCCCCCCGCCACCGCCGCCGCCGGCGGCCCCCCCCACCCCCAGCTGAACAAGAGCCAGAGCCTGACCAACGCCTTCAACCTGCCCGAGCCCGCCCCCCCCCGCCCCAGCCTGAGCCAGGACGAGGTGAAGGCCGAGACCATCCGCAGCCTGCGCAAGAGCTTCGCCAGCCTGTTCAGCGACTGA

hsSNCA | mammalian | this study

ATGGATGTATTCATGAAAGGACTTTCAAAGGCCAAGGAGGGAGTTGTGGCTGCTGCTGAGAAAACCAAACAGGGTGTGGCAGAAGCAGCAGGAAAGACAAAAGAGGGTGTTCTCTATGTAGGCTCCAAAACCAAGGAGGGAGTGGTGCATGGTGTGGCAACAGTGGCTGAGAAGACCAAAGAGCAAGTGACAAATGTTGGAGGAGCAGTGGTGACGGGTGTGACAGCAGTAGCCCAGAAGACAGTGGAGGGAGCAGGGAGCATTGCAGCAGCCACTGGCTTTGTCAAAAAGGACCAGTTGGGCAAGAATGAAGAAGGAGCCCCACAGGAAGGAATTCTGGAAGATATGCCTGTGGATCCTGACAATGAGGCTTATGAAATGCCTTCTGAGGAAGGGTATCAAGACTACGAACCTGAAGCCTAA

mCherry-Empty | mammalian | kind gift of Pietro De Camilli

ATGGGCCATCACCATCACCATCACAGCGGCAGCGAGAACCTGTACTTCCAGGGCGTGAGCAAGGGCGAGGAGGATAACATGGCCATCATCAAGGAGTTCATGCGCTTCAAGGTGCACATGGAGGGCTCCGTGAACGGCCACGAGTTCGAGATCGAGGGCGAGGGCGAGGGCCGCCCCTACGAGGGCACCCAGACCGCCAAGCTGAAGGTGACCAAGGGTGGCCCCCTGCCCTTCGCCTGGGACATCCTGTCCCCTCAGTTCATGTACGGCTCCAAGGCCTACGTGAAGCACCCCGCCGACATCCCCGACTACTTGAAGCTGTCCTTCCCCGAGGGCTTCAAGTGGGAGCGCGTGATGAACTTCGAGGACGGCGGCGTGGTGACCGTGACCCAGGACTCCTCCCTGCAGGACGGCGAGTTCATCTACAAGGTGAAGCTGCGCGGCACCAACTTCCCCTCCGACGGCCCCGTAATGCAGAAGAAGACCATGGGCTGGGAGGCCTCCTCCGAGCGGATGTACCCCGAGGACGGCGCCCTGAAGGGCGAGATCAAGCAGAGGCTGAAGCTGAAGGACGGCGGCCACTACGACGCTGAGGTCAAGACCACCTACAAGGCCAAGAAGCCCGTGCAGCTGCCCGGCGCCTACAACGTCAACATCAAGTTGGACATCACCTCCCACAACGAGGACTACACCATCGTGGAACAGTACGAACGCGCCGAGGGCCGCCACTCCACCGGCGGCATGGACGAGCTGTACAAG

SYP-FL | mammalian | this study

ATGGACGTGGTGAATCAGCTGGTGGCTGGGGGTCAGTTCCGGGTGGTCAAGGAGCCCCTTGGCTTCGTGAAGGTGCTGCAGTGGGTCTTTGCCATCTTCGCCTTTGCTACGTGCGGCAGCTACACCGGAGAGCTTCGGCTGAGCGTGGAGTGTGCCAACAAGACGGAGAGTGCCCTCAACATCGAAGTCGAATTTGAGTACCCATTCAGGCTGCACCAAGTGTACTTTGATGCACCCTCCTGCGTTAAAGGGGGCACTACCAAGATCTTCCTAGTTGGTGACTACTCCTCCTCGGCTGAATTCTTTGTCACCGTGGCTGTGTTTGCCTTCCTCTACTCCATGGGGGCCCTGGCCACCTACATCTTCCTGCAGAACAAGTACCGAGAGAACAACAAAGGGCCAATGATGGACTTCCTGGCCACAGCAGTGTTCGCTTTCATGTGGCTAGTTAGCTCATCCGCCTGGGCCAAAGGCCTGTCCGATGTGAAGATGGCCACTGACCCAGAGAACATTATCAAGGAGATGCCTATGTGCCGCCAGACAGGAAACACATGCAAGGAACTGAGGGACCCTGTGACTTCAGGACTCAACACCTCGGTGGTGTTTGGCTTCCTGAACCTGGTGCTCTGGGTTGGCAACCTATGGTTCGTGTTCAAGGAGACAGGCTGGGCCGCCCCATTCATGCGCGCACCTCCAGGCGCCCCAGAAAAGCAACCAGCTCCTGGCGATGCCTACGGCGATGCGGGCTATGGGCAGGGCCCCGGAGGCTATGGGCCCCAGGACTCCTACGGGCCTCAGGGTGGTTATCAACCCGATTACGGGCAGCCAGCCAGCGGCGGTGGCGGTGGCTACGGGCCTCAGGGCGACTATGGGCAGCAAGGCTACGGCCAACAGGGTGCGCCCACCTCCTTCTCCAATCAGATG

SYP-FL-EGFP | mammalian | kind gift of Pietro De Camilli

ATGGACGTGGTGAATCAGCTGGTGGCTGGGGGTCAGTTCCGGGTGGTCAAGGAGCCCCTTGGCTTCGTGAAGGTGCTGCAGTGGGTCTTTGCCATCTTCGCCTTTGCTACGTGCGGCAGCTACACCGGAGAGCTTCGGCTGAGCGTGGAGTGTGCCAACAAGACGGAGAGTGCCCTCAACATCGAAGTCGAATTTGAGTACCCATTCAGGCTGCACCAAGTGTACTTTGATGCACCCTCCTGCGTTAAAGGGGGCACTACCAAGATCTTCCTAGTTGGTGACTACTCCTCCTCGGCTGAATTCTTTGTCACCGTGGCTGTGTTTGCCTTCCTCTACTCCATGGGGGCCCTGGCCACCTACATCTTCCTGCAGAACAAGTACCGAGAGAACAACAAAGGGCCAATGATGGACTTCCTGGCCACAGCAGTGTTCGCTTTCATGTGGCTAGTTAGCTCATCCGCCTGGGCCAAAGGCCTGTCCGATGTGAAGATGGCCACTGACCCAGAGAACATTATCAAGGAGATGCCTATGTGCCGCCAGACAGGAAACACATGCAAGGAACTGAGGGACCCTGTGACTTCAGGACTCAACACCTCGGTGGTGTTTGGCTTCCTGAACCTGGTGCTCTGGGTTGGCAACCTATGGTTCGTGTTCAAGGAGACAGGCTGGGCCGCCCCATTCATGCGCGCACCTCCAGGCGCCCCAGAAAAGCAACCAGCTCCTGGCGATGCCTACGGCGATGCGGGCTATGGGCAGGGCCCCGGAGGCTATGGGCCCCAGGACTCCTACGGGCCTCAGGGTGGTTATCAACCCGATTACGGGCAGCCAGCCAGCGGCGGTGGCGGTGGCTACGGGCCTCAGGGCGACTATGGGCAGCAAGGCTACGGCCAACAGGGTGCGCCCACCTCCTTCTCCAATCAGATGTCGGATCCACCGGTCGCCACCATGGTGAGCAAGGGCGAGGAGCTGTTCACCGGGGTGGTGCCCATCCTGGTCGAGCTGGACGGCGACGTAAACGGCCACAAGTTCAGCGTGTCCGGCGAGGGCGAGGGCGATGCCACCTACGGCAAGCTGACCCTGAAGTTCATCTGCACCACCGGCAAGCTGCCCGTGCCCTGGCCCACCCTCGTGACCACCCTGACCTACGGCGTGCAGTGCTTCAGCCGCTACCCCGACCACATGAAGCAGCACGACTTCTTCAAGTCCGCCATGCCCGAAGGCTACGTCCAGGAGCGCACCATCTTCTTCAAGGACGACGGCAACTACAAGACCCGCGCCGAGGTGAAGTTCGAGGGCGACACCCTGGTGAACCGCATCGAGCTGAAGGGCATCGACTTCAAGGAGGACGGCAACATCCTGGGGCACAAGCTGGAGTACAACTACAACAGCCACAACGTCTATATCATGGCCGACAAGCAGAAGAACGGCATCAAGGTGAACTTCAAGATCCGCCACAACATCGAGGACGGCAGCGTGCAGCTCGCCGACCACTACCAGCAGAACACCCCCATCGGCGACGGCCCCGTGCTGCTGCCCGACAACCACTACCTGAGCACCCAGTCCGCCCTGAGCAAAGACCCCAACGAGAAGCGCGATCACATGGTCCTGCTGGAGTTCGTGACCGCCGCCGGGATCACTCTCGGCATGGACGAGCTGTACAAGTAA

mScarlet-mmSYNAPSIN 1 | mammalian | this study

ATGGTGAGCAAGGGCGAGGCAGTGATCAAGGAGTTCATGCGGTTCAAGGTGCACATGGAGGGCTCCATGAACGGCCACGAGTTCGAGATCGAGGGCGAGGGCGAGGGCCGCCCCTACGAGGGCACCCAGACCGCCAAGCTGAAGGTGACCAAGGGTGGCCCCCTGCCCTTCTCCTGGGACATCCTGTCCCCTCAGTTCATGTACGGCTCCAGGGCCTTCATCAAGCACCCCGCCGACATCCCCGACTACTATAAGCAGTCCTTCCCCGAGGGCTTCAAGTGGGAGCGCGTGATGAACTTCGAGGACGGCGGCGCCGTGACCGTGACCCAGGACACCTCCCTGGAGGACGGCACCCTGATCTACAAGGTGAAGCTCCGCGGCACCAACTTCCCTCCTGACGGCCCCGTAATGCAGAAGAAGACAATGGGCTGGGAAGCGTCCACCGAGCGGTTGTACCCCGAGGACGGCGTGCTGAAGGGCGACATTAAGATGGCCCTGCGCCTGAAGGACGGCGGCCGCTACCTGGCGGACTTCAAGACCACCTACAAGGCCAAGAAGCCCGTGCAGATGCCCGGCGCCTACAACGTCGACCGCAAGTTGGACATCACCTCCCACAACGAGGACTACACCGTGGTGGAACAGTACGAACGCTCCGAGGGCCGCCACTCCACCGGCGGCATGGACGAGCTGTACAAGTCCGGACTCAGATCTATGAACTACCTGCGGCGCCGCCTGTCGGACAGCAACTTCATGGCCAATCTGCCGAATGGGTACATGACAGACCTGCAGCGCCCGCAACCGCCCCCGCCGCCTCCCTCGGCCGCCAGCCCTGGGGCCACGCCCGGCTCCGCGACAGCCTCTGCCGAGAGGGCCTCCACAGCTGCTCCAGTGGCTTCTCCAGCAGCCCCTAGTCCTGGGTCCTCGGGGGGCGGCGGCTTCTTCTCGTCGCTGTCTAACGCGGTCAAGCAAACCACAGCAGCCGCAGCCGCCACCTTCAGCGAGCAGGTGGGCGGTGGCTCTGGGGGCGCAGGCCGCGGGGGCGCCGCCGCCAGGGTGCTGCTGGTCATCGACGAACCGCACACCGACTGGGCAAAATACTTCAAAGGGAAGAAGATCCATGGAGAAATTGACATTAAAGTAGAGCAAGCTGAATTCTCTGATCTCAATCTTGTGGCTCATGCCAATGGTGGATTCTCTGTGGACATGGAAGTTCTTCGGAATGGAGTCAAAGTTGTGAGGTCTCTGAAGCCAGACTTTGTGCTGATCCGCCAGCATGCCTTCAGCATGGCACGTAATGGAGACTACCGAAGTTTGGTCATTGGGCTGCAGTATGCTGGAATCCCCAGTGTAAACTCTTTGCATTCTGTCTACAACTTCTGTGACAAACCCTGGGTGTTTGCCCAGATGGTTCGACTACACAAGAAGCTTGGAACAGAGGAATTCCCTCTGATTGATCAGACTTTCTATCCTAATCACAAAGAGATGCTCAGCAGCACAACATACCCTGTGGTTGTGAAGATGGGCCACGCACATTCTGGGATGGGCAAGGTCAAGGTAGACAACCAACATGACTTCCAGGATATTGCAAGTGTTGTGGCACTGACTAAGACATATGCCACTGCTGAGCCCTTCATTGATGCTAAATATGATGTGCGTGTCCAGAAGATTGGGCAGAACTACAAGGCCTACATGAGGACATCAGTGTCGGGTAACTGGAAGACCAATACAGGTTCTGCTATGCTTGAGCAGATTGCCATGTCTGACAGGTACAAGTTGTGGGTAGACACGTGCTCAGAGATTTTTGGGGGACTTGACATCTGCGCAGTGGAAGCGCTGCATGGCAAGGACGGAAGGGATCACATTATTGAGGTGGTGGGCTCCTCCATGCCACTCATTGGTGATCACCAGGATGAAGACAAGCAGCTCATCGTGGAACTTGTGGTCAACAAGATGACTCAGGCTCTGCCTCGGCAGCCGCAGCGGGATGCTTCCCCTGGCAGGGGCTCCCACAGCCAGTCTTCATCCCCAGGAGCCCTGACCTTGGGCCGCCAGACCTCCCAGCAGCCTGCAGGTCCTCCTGCTCAACAACGACCCCCACCCCAGGGAGGCCCTCCACAGCCAGGCCCAGGACCTCAGCGCCAGGGACCCCCGCTGCAGCAGCGCCCACCCCCACAAGGCCAGCAACATCTTTCTGGCCTTGGACCGCCAGCTGGCAGCCCTCTGCCTCAGCGCCTACCAAGTCCCACCGCAGCACCTCAGCAGTCTGCCTCTCAGGCCACACCAGTGACCCAGGGTCAAGGCCGCCAGTCGCGGCCAGTGGCAGGAGGCCCTGGAGCACCTCCAGCAGCGCGCCCACCAGCCTCCCCATCTCCACAGCGTCAGGCGGGGGCCCCGCAGGCTACCCGTCAGGCATCTATCTCTGGTCCAGCTCCAACGAAGGCCTCAGGAGCCCCACCCGGAGGGCAGCAGCGCCAGGGCCCTCCCCAAAAACCCCCAGGCCCTGCTGGTCCCACTCGTCAGGCCAGTCAGGCAGGTCCCGGACCTCGCACTGGGCCTCCCACCACACAGCAGCCCCGGCCCAGCGGCCCAGGTCCTGCTGGACGTCCCGCCAAACCACAGCTGGCCCAGAAACCCAGCCAGGATGTGCCACCACCCATCACCGCCGCTGCCGGGGGACCCCCGCACCCCCAGCTCAACAAATCCCAGTCTCTGACCAATGCCTTCAACCTTCCAGAGCCAGCCCCTCCCAGGCCCAGCCTTAGCCAGGACGAGGTGAAAGCTGAGACCATCCGCAGCCTGAGGAAGTCTTTCGCCAGCCTCTTCTCCGACTGA

pET28-hsSNCA | *E. coli |* Wallace et al., 2024

ATGGATGTATTCATGAAAGGACTTTCAAAGGCCAAGGAGGGAGTTGTGGCTGCTGCTGAGAAAACCAAACAGGGTGTGGCAGAAGCAGCAGGAAAGACAAAAGAGGGTGTTCTCTATGTAGGCTCCAAAACCAAGGAGGGAGTGGTGCATGGTGTGGCAACAGTGGCTGAGAAGACCAAAGAGCAAGTGACAAATGTTGGAGGAGCAGTGGTGACGGGTGTGACAGCAGTAGCCCAGAAGACAGTGGAGGGAGCAGGGAGCATTGCAGCAGCCACTGGCTTTGTCAAAAAGGACCAGTTGGGCAAGAATGAAGAAGGAGCCCCACAGGAAGGAATTCTGGAAGATATGCCTGTGGATCCTGACAATGAGGCTTATGAAATGCCTTCTGAGGAAGGGTATCAAGACTACGAACCTGAAGCCTAA

pET30b-2H10-mCherry | *E. coli |* Geisterfer&Jalihal et al., 2024

ATGCACCATCATCATCATCATTCTTCTGGTGAAAACCTGTATTTTCAGGGCGCCGAGGTGCAGCTGGTGGAATCTGGGGGCGGACTGGTGCAGCCCGGGGGATCTCTGCGGCTGTCCTGCGCCGCCTCTGGCTCCATCTCTAGCGTGGATGTGATGTCCTGGTACAGGCAGGCCCCTGGCAAGCAGAGGGAACTGGTGGCCTTCATTACTGATAGGGGAAGAACCAATTACAAAGTGAGCGTGAAAGGCCGCTTCACTATCAGCCGGGATAATTCTAAGAATATGGTGTATCTGCAGATGAACAGCCTGAAGCCAGAAGACACTGCCGACTATCTGTGCAGGGCTGAGTCCCGGACTTCCTGGTCCAGCCCATCTCCTCTGGATGTGTGGGGGCGCGGCACTCAGGTGACTGTGTCCTCCGAATTGGATCCAGGTGGAGGTGGAAGCGGTGTGAGCAAGGGCGAGGAGGATAACATGGCCATCATCAAGGAGTTCATGCGCTTCAAGGTGCACATGGAGGGCTCCGTGAACGGCCACGAGTTCGAGATCGAGGGCGAGGGCGAGGGCCGCCCCTACGAGGGCACCCAGACCGCCAAGCTGAAGGTGACCAAGGGTGGCCCCCTGCCCTTCGCCTGGGACATCCTGTCCCCTCAGTTCATGTACGGCTCCAAGGCCTACGTGAAGCACCCCGCCGACATCCCCGACTACTTGAAGCTGTCCTTCCCCGAGGGCTTCAAGTGGGAGCGCGCGATGAACTTCGAGGACGGCGGCGTGGTGACCGTGACCCAGGACTCCTCCCTGCAGGACGGCGAGTTCATCTACAAGGTGAAGCTGCGCGGCACCAACTTCCCCTCCGACGGCCCCGTAATGCAGAAGAAGACCATGGGCTGGGAGGCCTCCTCCGAGCGGATGTACCCCGAGGACGGCGCCCTGAAGGGCGAGATCAAGCAGAGGCTGAAGCTGAAGGACGGCGGCCACTACGACGCTGAGGTCAAGACCACCTACAAGGCCAAGAAGCCCGTGCAGCTGCCCGGCGCCTACAACGTCAACATCAAGTTGGACATCACCTCCCACAACGAGGACTACACCATCGTGGAACAGTACGAACGCGCCGAGGGCCGCCACTCCACCGGCGGCATGGACGAGCTGTACAAGTAA

IVT Templates: expression system-[T7-promoter-cDNA] | source

pJet1.2-cln3.657 | this study

TAATACGACTCACTATAGGGAGAGTCTGCATACCAAAGATCAGCCGCTTGCATCACATGAGACCCTGCGGCACTCTCTTATTTTGCGTAGTTTCTTGGCCAAACTTAGGTAACCCACACTAAGAGGACACTGCCTGCATAAATACTGAAATTCTCTGTTCTCATCATCCGAGCAGTTAATAACTCCTAACGGACCCCATTTGCATCTTATTCGCACATCTGCGAAAGACTCTAGGAAACACCCACGCTCTAAAGCATCAATACTCCTATTGTCCGTAAATGGTACATCCGTGTGGGAAAGTCACGCGCTCCATCGCTGGTTCCAGACCAGAACGATGGAAGCGCATCAATATGCTTGCCAGCATGCCAGTGTTGCACTCCGCAAGCATCTCAGTGATCTGGAGAACCAGAAGCGGGCGTCCGGGACTGGCTTCCCGTACCTGCCGCAACTAGGTATCGGGATGTTCAACTTGAGTTTGCGCCACTTCATGTCCTTCCAAACTCGCTTGCGACTGTCCTAATCCACTCGCCTATCATGGCAAGACATCATGGGGCTTTTCAGCTCCAAGATGGTTTTTACTTCTACCCCCTTTCACCTACATACGCTTACGGCGGTAAGCCTGTAGTGATAGTTCTTACATAAAAACACCCGCACCCCGAGCCCGCAGCGCAATTGCTGCGACAACCGCTCACATGGGCGGTACCATGACCCGGAGCTGACCAACTTCTAGACGCTTCCCTGACCTGCGTATTCAGCTCCGAGTCATGTTTGCGCGCTTCATAACCTACAGATAACCGAGACCCCCAAGCCGTGCTCCATGCTCCTTAATCGAAACAGTTGTGAGCATGGAGCAGGCATAATTGGGTCAGTATGCAGCTCTTACCCAGGAGTTTACTATCATAACTGCTCCAACGCCATTCATATGGCGAGCGTTACCCCTATAACAATCGCATCCCGATATGCTGACCCCAAAGGGTCAGCATGCTGCAGGCCGTGTTTAACAGATGGAAATCTCATGGCGGTCTGCAGCCCCCATCTTTGCCTACAGGCCTGCGATGTCAAATTCCGGGCCTGTCTTTCCCTCTAGTCGGGATCCTGGAGCAGCGTGCGAATCTAAAGCACGACTATGTCTCCTCGGTAACCTTAATACCGCCAGTTAGCCGATGAGATCAATCTGTAGTATGGTTATGCAAGCGCTTCCGTGGGCTCGCCAAGTGCGGGTCTAGCTCCGGCCACTGCGGCCAGCGGCTCTCCCGCTGTCCGCATGGCAGGCAGCTAGACCCCGGCCTATGTCCCGACATCCGCTCTGGTGACCCTTACGCGGCATATGCCGACGATCAAGGCTTTTATGCCGCTCACTCCAACTACTTCTCGCAAGCCAAAGCGCCTAGTGCTCTGGCTTAGTGAAAAGTACCAGACGAGGCCCGCGCCTGTGGAGGTTACATCATAGGCGCGCGCAAAAGGCACTACCCATCAACATTCACCGCCTCTCCAGGAGATCGCGCAGTTGCTGCATCAGTAGCAACATCAGCGCGACCCCATGGCCGTGGACCTCCAGTTCAATGCCCGGGAACCACTCCTGGTCAAGAAACTACGCTAGCATAC

pJet1.2-cln3.362 | this study

TAATACGACTCACTATAGGGAGAGTCTGCATACCAAAGATCAGCCGCTTGCATTGAAAGGGACGAACGGTGGCTCATGTCTTTGCCATCCTTTCTTCTCCAATCCCCGAAGCACTTCAGACACACACGTAACCTTGCATGAAATATAGTATTTATTGTCATCGCCGGCTGGACTGTTACTATCTCAAGCCCAAAGACCATTGCATATCACACACAAATTCGTCACAAACACTTAAAGATTGTCCTGCGGTCAGGCTTCTCTGCTACACCGGGCCTCTGTCAGTATGTCTGTTTGGGAACGTAAGTGTTCGCATCACCGGCTAATAAAAGTGGACGTGTTTGCGTAACAAACTGACTGCTCGGAGAACCGCATTTTAACCCTTACGCTTCTGATTGATCTCGTGTATCACACCCGGTAATCATGTACTTGTTTCCAGTCCCGACCCTTAGTTACCGTGGCGATTCGCAAATTGATTGATGATTCTTGCCCTAGTTTCCCGCCATCCTTGTGCTTCCCCTCTTCGACGCTCTTTATAGGCAGCGCCAAATTTTATCGGTAGCGCGCTATGTTCATTGTTATGTCACCCACATATCTTCTTCTTGGGCTTGACACTATTTGGCTGACGTCCAAGTTCGCAGATCTAATGCCCCGACTATCGAGCTTCCAAAGCCTCCGCCGCCACGAGCAAAAGAAACCGCATTTCACGACATCAGAGCCGACAATCCCCAACACCCTTCACTGGTCAGTATGTACTACGGCGACTCTCTATTCATAGGCGGAGATCAACCGGCAAACAAAGATAGGCAGTCTCCAGTTCTGGCCCATCTCTATGAGCAGTTTAAGGTCTCGAGCACCGAATCTTTGCAAGATATTCCCGTATGACCCAGACCCGAAATACAATACCAAGTTCAGCGCCGTTCACTTCGCAAGCGTAAGAGTTCTAAAGACCGTACGCAGGCTCGCTGTGCTCTCCTAGGTCATGGTTTGCTGACGATGTACAACTGACAAAAATCCCATTCCCATCCGCACCCAGGTTCTAGAGGTAGTCGCCAACCCGTCCAACGCAAACTCCAGATCTCCACCCAATTGCCTTTCAGGTGGTGCCTCGATCCTTGCCGTCTAAATTATAGCCAGCCTGTGCCCATTTAACCGACGGTTAAGTAATGAGAAAAAGCGTTCCTGGCGAGATGCAGGCACGGGGGTGGGAATCGAATGGCCAGGCGCCGCAGTGCTCCGTGCTACCTGCCTCTCCCCCGATATCAGCAGTGACGGCGACTCTAGCTCGGGCTCTGTTCCACCATTTACTCTGGACCGGCCCGCCCCGCACAAGACGACACTCAAGACAGCTGTGGCCCCCCTAACATCTACTCCGAACGACTCACCGCGCACTCATCTCACGTATCGTTCGATGCCCCAGACGCCACCCACTATTATGTCCGTTGCAGCTCAGGCGCGTGCACAATGCCTCCCAGACCTACACTCCACCACGACTAAGAACCCCCTTCACTTGCTGCATTCTGAAGAATCTAAGGACTCCGCCACGGGTATCGACCATGAGCTATGCGACATTGTTCAGGACTCGGTCAAGAAACTACGCTAGCATAC

pJet1.2-atp5b | this study

TAATACGACTCACTATAGGTCTCCACCCGGATTCCGCCATGTTGAGTCTTGTGGGGCGTGTGGCCTCGGCCTCGGCCTCCGGGGCCTTGCGGGGACTCAGCCCTTCGGCGGCTCTGCCACAGGCGCAGCTTCTACTGCGAGCTGCTCCCGCCGGGGTTCATCCTGCCAGAGACTATGCGGCGCAGGCGTCTGCGGCCCCGAAGGCAGGCACTGCCACCGGGCGAATCGTGGCAGTCATCGGCGCTGTGGTGGACGTCCAGTTCGATGAGGGATTACCACCCATCCTAAATGCCCTGGAAGTGCAAGGCAGGGACAGCAGACTGGTTTTGGAGGTGGCCCAGCATTTGGGGGAGAGCACGGTCAGAACTATTGCTATGGATGGCACTGAAGGCTTGGTTAGAGGCCAGAAAGTACTGGATTCAGGGGCACCAATCAAAATTCCTGTTGGTCCTGAGACCTTGGGCAGAATCATGAATGTCATTGGAGAACCTATTGATGAGAGAGGTCCTATCAAAACCAAACAATTTGCTCCTATTCATGCTGAGGCTCCTGAGTTCATAGAGATGAGTGTTGAGCAGGAGATTCTGGTGACTGGGATAAAGGTTGTGGATCTGCTGGCCCCATACGCCAAGGGTGGGAAAATCGGACTCTTTGGAGGTGCTGGCGTTGGAAAGACAGTACTGATCATGGAGCTAATCAACAATGTCGCCAAAGCCCATGGTGGTTACTCTGTATTTGCTGGTGTTGGTGAGAGGACCCGTGAGGGCAATGATTTATACCATGAAATGATTGAATCTGGTGTTATCAATCTAAAAGATGCCACTTCCAAGGTAGCGTTGGTATATGGACAGATGAACGAACCACCTGGCGCTCGAGCCCGGGTAGCTCTGACTGGTTTGACCGTTGCTGAATACTTCAGAGACCAGGAGGGCCAAGATGTCCTGCTGTTTATTGACAACATCTTCCGCTTTACCCAGGCTGGCTCAGAGGTGTCTGCCTTATTGGGCAGAATCCCTTCTGCTGTAGGCTACCAGCCCACCCTAGCCACCGACATGGGCACAATGCAGGAAAGGATCACCACCACCAAGAAGGGATCGATCACCTCGGTGCAGGCTATCTATGTGCCTGCTGATGACCTGACTGACCCTGCCCCTGCAACCACCTTTGCCCATTTGGATGCTACCACTGTGTTGTCCCGGGCTATTGCTGAGTTGGGCATCTATCCAGCTGTGGATCCACTGGACTCCACCTCTCGAATTATGGATCCCAACATTGTTGGCAATGAGCATTATGACGTCGCCCGAGGAGTGCAGAAAATCCTGCAGGACTACAAATCTCTCCAGGACATCATTGCCATCTTGGGTATGGATGAACTTTCTGAGGAAGATAAATTGACTGTGTCCCGGGCAAGAAAGATACAGCGCTTCTTGTCTCAGCCATTCCAAGTTGCTGAGGTCTTCACGGGTCACATGGGGAAGCTGGTGCCCTTGAAGGAGACCATTAAAGGATTCCAGCAGATTTTAGCAGGTGAATATGACCATCTCCCAGAACAAGCCTTCTACATGGTGGGACCCATTGAAGAAGCCGTGGCAAAGGCTGATAAGCTGGCAGAAGAGCACGGGTCGTGAGGGACTCCAGCCAAAGGCAGCACTGCAACTGATCTCTCCATATCAAGCGAGAGCTCAGGTTTCCTTCCATGCAGGCCACACAAGAGCCTTGATTGAAGATGTGATGTTCTCTCTGAAGAGTATTTAAAGTTTTCAATAAAGTATATACCCCTCATTTATGTCTGTTTATGTTGCTTCTGAAACAGCTTATAATTGAGCTCACAGTGGTGGGCCACATTTAGGAAATTGTTAGAAACAGGATTATTATTAAAAAGATCAATTTATTAATG

pJet1.2-scg3 | this study

TAATACGACTCACTATAGGGAGCATGCGCCTCCTCCTCATTTCCTCTCCTGGAGCTAGTGGAGTAAAGCTACGCCCAGGCCCGCGTCCGCTGGCGGCGCAGGAACTTCAGCACCCGCGGGGCGGACAGCGCCTACCGCACCTGCTCACCTGCTCTGGGCGCCAGAAGAGCCTGCATCCTCCTTCCAGCCCGGAGCAACTGCGCCGGAGGCGCCCAGACCCTCTCCCTTCCCGCACCCAGGCTCCTGTCCCTTCCAGCTTCTTAACTCCCCTTCTCATTCATAACAAAAGCTACAGCTCAGGGGCCCAGCGCCAAGCTCTTTCCAGCAAAGCACAGAAGAGCAAGAAAGAATGGGGTTCCTTTGGACCGGCTCTTGGATACTGGTGTTGGTGCTCAACAGCGGCCCAATTCAAGCTTTCCCCAAACCCGAAGGCAGCCAAGACAAATCCCTGCATAATAGAGAATTAAGTGCAGAAAGACCTTTGAATGAACAGATCGCTGAGGCAGAGGCAGACAAGATTAAAAAGGCATTCCCTTCAGAAAGCAAGCCGAGTGAAAGCAATTATTCTTCTGTCGATAACTTGAATCTGCTGAGGGCAATAACAGAAAAGGAAACCGTTGAGAAAGAGAGACAATCCATAAGAAGCCCCCCGTTTGATAACCAACTGAACGTGGAAGACGCTGATTCAACCAAAAATCGGAAACTGATCGATGAGTACGATTCCACCAAGAGTGGACTGGACCACAAGTTTCAAGATGACCCAGACGGCCTTCATCAACTGGATGGAACTCCTTTAACTGCTGAAGACATCGTCCATAAGATTGCCACCAGGATTTATGAGGAGAACGACAGAGGAGTGTTTGACAAAATTGTTTCTAAACTGCTGAATCTTGGCCTGATCACTGAAAGCCAGGCACATACTCTGGAAGATGAAGTAGCAGAAGCTTTACAAAAACTGATTTCAAAAGAGGCCAACAATTATGAGGAGACCCTGGATAAACCCACAAGCAGGACCGAGAATCAGGATGGGAAAATACCAGAGAAAGTGACTCCGGTGGCAGCAGTCCAAGATGGCTTCACTAACCGTGAAAACGATGAGACGGTGTCTAACACCTTGACCTTGTCCAATGGCTTGGAAAGGAGAACTAACCCCCACAGGGAAGACGACTTTGAGGAACTCCAGTATTTCCCCAACTTCTATGCACTACTGACAAGCATCGACTCAGAAAAAGAAGCAAAAGAGAAAGAAACCCTGATCACCATCATGAAGACATTGATTGACTTCGTGAAAATGATGGTGAAATACGGTACGATATCTCCAGAGGAAGGCGTGTCCTACCTTGAAAACTTGGATGAAACAATTGCTCTGCAGACCAAGAACAAGCTAGAAAAAAATACTACTGATAGCAAAACTCCACCAGAGAAGAGTCAGGAAGAAACAGACAGTACCAAGGAAGAAGCCGCCAAGATGGAAAAGGAATACGGAAGCCTAAAAGACTCTACAAAAGATGATAACTCCAACCTAGGAGGAAAGACAGATGAAGCCACAGGGAAGACAGAAGCCTACTTGGAAGCCATTAGAAAAAACATCGAATGGCTGAAGAAACATAACAAGAAGGGCAACAAAGAAGATTACGACCTTTCAAAGATGAGGGACTTTATCAACCAACAAGCTGACGCTTATGTGGAGAAGGGCATCCTCGACAAGGAAGAAGCCAACGCCATCAAACGCATCTACAGCAGCCTGTGAAAATGGCGGGCAGCTTGAGCCTTCCTGTTGTTCCAGCAAAAACAATATAGCTTACAAACTAATTCGGCGGTTAAAGGGTTACCAGCCCAGAAGTATTAGGATGTGCTGAATTTATAGTAGTTAATCCCTTAGAAATGAGTAAAATAGAGCTCTCTTGCCATAAATACCTTATGAAAAGCAAAGCTGTAGAGAAGCCGAGGTTTTTCTATATAGAATCCTTATTTCCTCTTGAATTTACATTTTGTAATCAGAGATGTGCTGCTCTGGAAAAGACTCTAATGGGTTGAACATAAGTCTGAACCTACTCCCCACTGTCCTCAGCCCCCTGAAGCTCTGAGAGGCCCTGTCTCGGCATGCTAGACACCTGAGCACCTCACTGGATGTTTGTCATAGGATGTCGTTTCCACTAGTCGATCTCTGTTGGGCACGGAAATAAACCCACGTCTCTTCATATCCTTGA

pJet1.2-prnp | this study

TAATACGACTCACTATAGCCCCTTTCCACTCCCGGCTCCCCCGCGTTGTCGGATCAGCAGACCGATTCTGGGCGCTGCGTCGCATCGGTGGCAGGACTCCTGAGTATATTTCAGAACTGAACCATTTCAACCGAGCTGAAGCATTCTGCCTTCCTAGTGGTACCAGTCCAATTTAGGAGAGCCAAGCAGACTATCAGTCATCATGGCGAACCTTGGCTACTGGCTGCTGGCCCTCTTTGTGACTATGTGGACTGATGTCGGCCTCTGCAAAAAGCGGCCAAAGCCTGGAGGGTGGAACACCGGTGGAAGCCGGTATCCCGGGCAGGGAAGCCCTGGAGGCAACCGTTACCCACCTCAGGGTGGCACCTGGGGGCAGCCCCACGGTGGTGGCTGGGGACAACCCCATGGGGGCAGCTGGGGACAACCTCATGGTGGTAGTTGGGGTCAGCCCCATGGCGGTGGATGGGGCCAAGGAGGGGGTACCCATAATCAGTGGAACAAGCCCAGCAAACCAAAAACCAACCTCAAGCATGTGGCAGGGGCTGCGGCAGCTGGGGCAGTAGTGGGGGGCCTTGGTGGCTACATGCTGGGGAGCGCCATGAGCAGGCCCATGATCCATTTTGGCAACGACTGGGAGGACCGCTACTACCGTGAAAACATGTACCGCTACCCTAACCAAGTGTACTACAGGCCAGTGGATCAGTACAGCAACCAGAACAACTTCGTGCACGACTGCGTCAATATCACCATCAAGCAGCACACGGTCACCACCACCACCAAGGGGGAGAACTTCACCGAGACCGATGTGAAGATGATGGAGCGCGTGGTGGAGCAGATGTGCGTCACCCAGTACCAGAAGGAGTCCCAGGCCTATTACGACGGGAGAAGATCCAGCAGCACCGTGCTTTTCTCCTCCCCTCCTGTCATCCTCCTCATCTCCTTCCTCATCTTCCTGATCGTGGGATGAGGGAGGCCTTCCTGCTTGTTCCTTCGCATTCTCGTGGTCTAGGCTGGGGGAGGGGTTATCCACCTGTAGCTCTTTCAATTGAGGTGGTTCTCATTCTTGCTTCTCTGTGTCCCCCATAGGCTAATACCCCTGGCACTGATGGGCCCTGGGAAATGTACAGTAGACCAGTTGCTCTTTGCTTCAGGTCCCTTTGATGGAGTCTGTCATCAGCCAGTGCTAACACCGGGCCAATAAGAATATAACACCAAATAACTGCTGGCTAGTTGGGGCTTTGTTTTGGTCTAGTGAATAAATACTGGTGTATCCCCTGACTTGTACCCAGAGTACAAGGTGACAGTGACACATGTAACTTAGCATAGGCAAAGGGTTCTACAACCAAAGAAGCCACTGTTTGGGGATGGCGCCCTGGAAAACAGCCTCCCACCTGGGATAGCTAGAGCATCCACACGTGGAATTCTTTCTTTACTAACAAACGATAGCTGATTGAAGGCAACAGGAAAAAAAAAATCAAATTGTCCTACTGACGTTGAAAGCAAACCTTTGTTCATTCCCAGGGCACTAGAATGATCTTTAGCCTTGCTTGGATTGAACTAGGAGATCTTGACTCTGAGGAGAGCCAGCCCTGTAAAAAGCTTGGTCCTCCTGTGACGGGAGGGATGGTTAAGGTACAAAGGCTAGAAACTTGAGTTTCTTCATTTCTGTCTCACAATTATCAAAAGCTAGAATTAGCTTCTGCCCTATGTTTCTGTACTTCTATTTGAACTGGATAACAGAGAGACAATCTAAACATTCTCTTAGGCTGCAGATAAGAGAAGTAGGCTCCATTCCAAAGTGGGAAAGAAATTCTGCTAGCATTGTTTAAATCAGGCAAAATTTGTTCCTGAAGTTGCTTTTTACCCCAGCAGACATAAACTGCGATAGCTTCAGCTTGCACTGTGGATTTTCTGTATAGAATATATAAAACATAACTTCAAGCTTATGTCTTCTTTTTAAAACATCTGAAGTATGGGACGCCCTGGCCGTTCCATCCAGTACTAAATGCTTACCGTGTGACCCTTGGGCTTTCAGCGTGCACTCAGTTCCGTAGGATTCCAAAGCAGACCCCTAGCTGGTCTTTGAATCTGCATGTACTTCACGTTTTCTATATTTGTAACTTTGCATGTATTTTGTTTTGTCATATAAAAAGTTTATAAATGTTTGCTATCAGACTGACATTAAATAGAAGCTATGATGAACACCTGG

pJet1.2-atp5b::nluc | this study

TAATACGACTCACTATAAGGCCACCATGTTGAGTCTTGTGGGGCGTGTGGCCTCGGCCTCGGCCTCCGGGGCCTTGCGGGGACTCAGCCCTTCGGCGGCTCTGCCACAGGCGCAGCTTCTACTGCGAGCTGCTCCCGCCGGGGTTCATCCTGCCAGAGACTATGCGGCGCAGGCGTCTGCGGCCCCGAAGGCAGGCACTGCCACCGGGCGAATCGTGGCAGTCATCGGCGCTGTGGTGGACGTCCAGTTCGATGAGGGATTACCACCCATCCTAAATGCCCTGGAAGTGCAAGGCAGGGACAGCAGACTGGTTTTGGAGGTGGCCCAGCATTTGGGGGAGAGCACGGTCAGAACTATTGCTATGGATGGCACTGAAGGCTTGGTTAGAGGCCAGAAAGTACTGGATTCAGGGGCACCAATCAAAATTCCTGTTGGTCCTGAGACCTTGGGCAGAATCATGAATGTCATTGGAGAACCTATTGATGAGAGAGGTCCTATCAAAACCAAACAATTTGCTCCTATTCATGCTGAGGCTCCTGAGTTCATAGAGATGAGTGTTGAGCAGGAGATTCTGGTGACTGGGATAAAGGTTGTGGATCTGCTGGCCCCATACGCCAAGGGTGGGAAAATCGGACTCTTTGGAGGTGCTGGCGTTGGAAAGACAGTACTGATCATGGAGCTAATCAACAATGTCGCCAAAGCCCATGGTGGTTACTCTGTATTTGCTGGTGTTGGTGAGAGGACCCGTGAGGGCAATGATTTATACCATGAAATGATTGAATCTGGTGTTATCAATCTAAAAGATGCCACTTCCAAGGTAGCGTTGGTATATGGACAGATGAACGAACCACCTGGCGCTCGAGCCCGGGTAGCTCTGACTGGTTTGACCGTTGCTGAATACTTCAGAGACCAGGAGGGCCAAGATGTCCTGCTGTTTATTGACAACATCTTCCGCTTTACCCAGGCTGGCTCAGAGGTGTCTGCCTTATTGGGCAGAATCCCTTCTGCTGTAGGCTACCAGCCCACCCTAGCCACCGACATGGGCACAATGCAGGAAAGGATCACCACCACCAAGAAGGGATCGATCACCTCGGTGCAGGCTATCTATGTGCCTGCTGATGACCTGACTGACCCTGCCCCTGCAACCACCTTTGCCCATTTGGATGCTACCACTGTGTTGTCCCGGGCTATTGCTGAGTTGGGCATCTATCCAGCTGTGGATCCACTGGACTCCACCTCTCGAATTATGGATCCCAACATTGTTGGCAATGAGCATTATGACGTCGCCCGAGGAGTGCAGAAAATCCTGCAGGACTACAAATCTCTCCAGGACATCATTGCCATCTTGGGTATGGATGAACTTTCTGAGGAAGATAAATTGACTGTGTCCCGGGCAAGAAAGATACAGCGCTTCTTGTCTCAGCCATTCCAAGTTGCTGAGGTCTTCACGGGTCACATGGGGAAGCTGGTGCCCTTGAAGGAGACCATTAAAGGATTCCAGCAGATTTTAGCAGGTGAATATGACCATCTCCCAGAACAAGCCTTCTACATGGTGGGACCCATTGAAGAAGCCGTGGCAAAGGCTGATAAGCTGGCAGAAGAGCACGGGTCGATGGTCTTCACACTCGAAGATTTCGTTGGGGACTGGCGACAGACAGCCGGCTACAACCTGGACCAAGTCCTTGAACAGGGAGGTGTGTCCAGTTTGTTTCAGAATCTCGGGGTGTCCGTAACTCCGATCCAAAGGATTGTCCTGAGCGGTGAAAATGGGCTGAAGATCGACATCCATGTCATCATCCCGTATGAAGGTCTGAGCGGCGACCAAATGGGCCAGATCGAAAAAATTTTTAAGGTGGTGTACCCTGTGGATGATCATCACTTTAAGGTGATCCTGCACTATGGCACACTGGTAATCGACGGGGTTACGCCGAACATGATCGACTATTTCGGACGGCCGTATGAAGGCATCGCCGTGTTCGACGGCAAAAAGATCACTGTAACAGGGACCCTGTGGAACGGCAACAAAATTATCGACGAGCGCCTGATCAACCCCGACGGCTCCCTGCTGTTCCGAGTAACCATCAACGGAGTGACCGGCTGGCGGCTGTGCGAACGCATTCTGGCGGGTGGGGGATCATTGTACAAATAA

pJet1.2-atp5b::moon | this study

TAATACGACTCACTATAAGGCCACCATGTTGAGTCTTGTGGGGCGTGTGGCCTCGGCCTCGGCCTCCGGGGCCTTGCGGGGACTCAGCCCTTCGGCGGCTCTGCCACAGGCGCAGCTTCTACTGCGAGCTGCTCCCGCCGGGGTTCATCCTGCCAGAGACTATGCGGCGCAGGCGTCTGCGGCCCCGAAGGCAGGCACTGCCACCGGGCGAATCGTGGCAGTCATCGGCGCTGTGGTGGACGTCCAGTTCGATGAGGGATTACCACCCATCCTAAATGCCCTGGAAGTGCAAGGCAGGGACAGCAGACTGGTTTTGGAGGTGGCCCAGCATTTGGGGGAGAGCACGGTCAGAACTATTGCTATGGATGGCACTGAAGGCTTGGTTAGAGGCCAGAAAGTACTGGATTCAGGGGCACCAATCAAAATTCCTGTTGGTCCTGAGACCTTGGGCAGAATCATGAATGTCATTGGAGAACCTATTGATGAGAGAGGTCCTATCAAAACCAAACAATTTGCTCCTATTCATGCTGAGGCTCCTGAGTTCATAGAGATGAGTGTTGAGCAGGAGATTCTGGTGACTGGGATAAAGGTTGTGGATCTGCTGGCCCCATACGCCAAGGGTGGGAAAATCGGACTCTTTGGAGGTGCTGGCGTTGGAAAGACAGTACTGATCATGGAGCTAATCAACAATGTCGCCAAAGCCCATGGTGGTTACTCTGTATTTGCTGGTGTTGGTGAGAGGACCCGTGAGGGCAATGATTTATACCATGAAATGATTGAATCTGGTGTTATCAATCTAAAAGATGCCACTTCCAAGGTAGCGTTGGTATATGGACAGATGAACGAACCACCTGGCGCTCGAGCCCGGGTAGCTCTGACTGGTTTGACCGTTGCTGAATACTTCAGAGACCAGGAGGGCCAAGATGTCCTGCTGTTTATTGACAACATCTTCCGCTTTACCCAGGCTGGCTCAGAGGTGTCTGCCTTATTGGGCAGAATCCCTTCTGCTGTAGGCTACCAGCCCACCCTAGCCACCGACATGGGCACAATGCAGGAAAGGATCACCACCACCAAGAAGGGATCGATCACCTCGGTGCAGGCTATCTATGTGCCTGCTGATGACCTGACTGACCCTGCCCCTGCAACCACCTTTGCCCATTTGGATGCTACCACTGTGTTGTCCCGGGCTATTGCTGAGTTGGGCATCTATCCAGCTGTGGATCCACTGGACTCCACCTCTCGAATTATGGATCCCAACATTGTTGGCAATGAGCATTATGACGTCGCCCGAGGAGTGCAGAAAATCCTGCAGGACTACAAATCTCTCCAGGACATCATTGCCATCTTGGGTATGGATGAACTTTCTGAGGAAGATAAATTGACTGTGTCCCGGGCAAGAAAGATACAGCGCTTCTTGTCTCAGCCATTCCAAGTTGCTGAGGTCTTCACGGGTCACATGGGGAAGCTGGTGCCCTTGAAGGAGACCATTAAAGGATTCCAGCAGATTTTAGCAGGTGAATATGACCATCTCCCAGAACAAGCCTTCTACATGGTGGGACCCATTGAAGAAGCCGTGGCAAAGGCTGATAAGCTGGCAGAAGAGCACGGGTCGATGCGTACGGGCAAGAATGAGCAGGAACTGCTGGAACTGGACAAATGGGCAAGCCTGGGGAGCGGCTCCGGAAAGAACGAGCAGGAACTGCTGGAACTGGACAAGTGGGCTTCTCTGGGCTCCGGGTCCGGGAAAAACGAACAGGAGCTGCTGGAGCTGGATAAGTGGGCCAGCCTGGGCTCCGGGTCCGGGAAGAATGAACAGGAACTGCTGGAACTGGATAAATGGGCCTCTCTGGGCAGCGGGTCCGGAAAGAATGAACAGGAGCTGCTGGAACTGGACAAGTGGGCGTCCCTGGGGAGCGGGTCCGGAAAGAATGAACAGGAACTGCTGGAACTGGATAAGTGGGCTTCCCTGGGAAGCGGCTCCGGAAAAAACGAGCAGGAGCTGCTGGAGCTGGACAAGTGGGCCTCTCTGGGCTCCGGCTCCGGGAAGAATGAACAGGAGCTGCTGGAACTGGATAAGTGGGCCTCTCTGGGGTCCGGAAGCGGTAAAAACGAGCAGGAACTGCTGGAACTGGACAAGTGGGCAAGCCTGGGGTCTGGCTCCGGCAAAAACGAACAGGAACTGCTGGAACTGGACAAATGGGCCAGCCTGGGCTCCGGGTCCGGGAAAAATGAGCAGGAACTGCTGGAACTGGATAAATGGGCGAGCCTGGGCAGCGGGTCCGGAAAAAACGAACAGGAGCTGCTGGAGCTGGACAAATGGGCGAGTCTGGGCTCCGGAAGCGGCAAGAATGAACAGGAACTGCTGGAGCTGGACAAATGGGCCAGCCTGGGCAGCGGCTCCGGAAAGAATGAGCAGGAACTGCTGGAGCTGGACAAATGGGCGTCTCTGGGCTCCGGCTCCGGGAAAAACGAGCAGGAACTGCTGGAGCTGGACAAGTGGGCTTCCCTGGGCAGCGGCTCCGGAAAAAATGAACAGGAACTGCTGGAACTGGACAAGTGGGCCAGCCTGGGGTCCGGGTCTGGGAAGAACGAGCAGGAACTGCTGGAGCTGGACAAGTGGGCTTCTCTGGGGTCTGGCTCTGGCAAGAACGAGCAGGAACTGCTGGAACTGGACAAGTGGGCGAGCCTGGGCAGCGGGTCTGGCAAGAATGAACAGGAGCTGCTGGAACTGGACAAATGGGCCAGCCTGGGGTCTGGGTCTGGAAAGAATGAGCAGGAACTGCTGGAGCTGGACAAGTGGGCCTCTCTGGGCTCTGGGTCTGGCAAGAACGAGCAGGAGCTGCTGGAGCTGGATAAGTGGGCGTCCCTGGGGTCCGGGTCCGGCAAGAATGAACAGGAGCTGCTGGAGCTGGATAAATGGGCTTCCCTGGGCTCTGGGTCTGGGAAGAATGAGCAGGAGCTGCTGGAACTGGATAAGTGGGCGAGCCTGGGCAGCGGGTCTGGCAAGAATGAGCAGGAACTGCTGGAGCTGGACAAGTGGGCTTCTCTGTAA

**Supplementary Table S2: cDNA and Primers for in vitro transcription.**

| Table S2 Primers for in vitro transcription | | |
| --- | --- | --- |
| Plasmid | **Forward primer** | **Reverse primer** |
| pJet1.2-cln3.657 | taatacgactcactataggg | gtatgctagcgtagtttctt |
| pJet1.2-cln3.362 | taatacgactcactataggg | gtatgctagcgtagtttctt |
| pJet1.2-atp5b | taatacgactcactataggtctccacccgg | cattaataaattgatctttttaataataatcctgtttctaacaatttcctaaatgtggcc |
| pJet1.2-scg3 | taatacgactcactatagggagcatgcgc | tcaaggatatgaagagacgtgggtttatttccg |
| pJet1.2-prnp | taatacgactcactatagcccctttccactcc | ccaggtgttcatcatagcttctatttaatgtcagtctg |
| pJet1.2-atp5b::nluc | taatacgactcactataaggccaccatgttgagtcttgtg | ttatttgtacaatgatcccccacccgc |
| pJet1.2-atp5b::moon | cDNA template was digested with HindIII to linearize prior to IVT | cDNA template was digested with HindIII to linearize prior to IVT |

**Supplementary Table S3: Sequences of** **synaptic and *CLN3* mRNAs used for in vitro reconstitutions.**

Table S3 Sequences of synaptic and *CLN3* mRNAs used for in vitro reconstitutions

Transcript sequence (5->3'))

*Atp5b* | NM_016774.3 | *Mus musculus*

GTCTCCACCCGGATTCCGCCATGTTGAGTCTTGTGGGGCGTGTGGCCTCGGCCTCGGCCTCCGGGGCCTTGCGGGGACTCAGCCCTTCGGCGGCTCTGCCACAGGCGCAGCTTCTACTGCGAGCTGCTCCCGCCGGGGTTCATCCTGCCAGAGACTATGCGGCGCAGGCGTCTGCGGCCCCGAAGGCAGGCACTGCCACCGGGCGAATCGTGGCAGTCATCGGCGCTGTGGTGGACGTCCAGTTCGATGAGGGATTACCACCCATCCTAAATGCCCTGGAAGTGCAAGGCAGGGACAGCAGACTGGTTTTGGAGGTGGCCCAGCATTTGGGGGAGAGCACGGTCAGAACTATTGCTATGGATGGCACTGAAGGCTTGGTTAGAGGCCAGAAAGTACTGGATTCAGGGGCACCAATCAAAATTCCTGTTGGTCCTGAGACCTTGGGCAGAATCATGAATGTCATTGGAGAACCTATTGATGAGAGAGGTCCTATCAAAACCAAACAATTTGCTCCTATTCATGCTGAGGCTCCTGAGTTCATAGAGATGAGTGTTGAGCAGGAGATTCTGGTGACTGGGATAAAGGTTGTGGATCTGCTGGCCCCATACGCCAAGGGTGGGAAAATCGGACTCTTTGGAGGTGCTGGCGTTGGAAAGACAGTACTGATCATGGAGCTAATCAACAATGTCGCCAAAGCCCATGGTGGTTACTCTGTATTTGCTGGTGTTGGTGAGAGGACCCGTGAGGGCAATGATTTATACCATGAAATGATTGAATCTGGTGTTATCAATCTAAAAGATGCCACTTCCAAGGTAGCGTTGGTATATGGACAGATGAACGAACCACCTGGCGCTCGAGCCCGGGTAGCTCTGACTGGTTTGACCGTTGCTGAATACTTCAGAGACCAGGAGGGCCAAGATGTCCTGCTGTTTATTGACAACATCTTCCGCTTTACCCAGGCTGGCTCAGAGGTGTCTGCCTTATTGGGCAGAATCCCTTCTGCTGTAGGCTACCAGCCCACCCTAGCCACCGACATGGGCACAATGCAGGAAAGGATCACCACCACCAAGAAGGGATCGATCACCTCGGTGCAGGCTATCTATGTGCCTGCTGATGACCTGACTGACCCTGCCCCTGCAACCACCTTTGCCCATTTGGATGCTACCACTGTGTTGTCCCGGGCTATTGCTGAGTTGGGCATCTATCCAGCTGTGGATCCACTGGACTCCACCTCTCGAATTATGGATCCCAACATTGTTGGCAATGAGCATTATGACGTCGCCCGAGGAGTGCAGAAAATCCTGCAGGACTACAAATCTCTCCAGGACATCATTGCCATCTTGGGTATGGATGAACTTTCTGAGGAAGATAAATTGACTGTGTCCCGGGCAAGAAAGATACAGCGCTTCTTGTCTCAGCCATTCCAAGTTGCTGAGGTCTTCACGGGTCACATGGGGAAGCTGGTGCCCTTGAAGGAGACCATTAAAGGATTCCAGCAGATTTTAGCAGGTGAATATGACCATCTCCCAGAACAAGCCTTCTACATGGTGGGACCCATTGAAGAAGCCGTGGCAAAGGCTGATAAGCTGGCAGAAGAGCACGGGTCGTGAGGGACTCCAGCCAAAGGCAGCACTGCAACTGATCTCTCCATATCAAGCGAGAGCTCAGGTTTCCTTCCATGCAGGCCACACAAGAGCCTTGATTGAAGATGTGATGTTCTCTCTGAAGAGTATTTAAAGTTTTCAATAAAGTATATACCCCTCATTTATGTCTGTTTATGTTGCTTCTGAAACAGCTTATAATTGAGCTCACAGTGGTGGGCCACATTTAGGAAATTGTTAGAAACAGGATTATTATTAAAAAGATCAATTTATTAATG

*Prnp* | NM_001164790.1 | *Mus musculus*

CCCCTTTCCACTCCCGGCTCCCCCGCGTTGTCGGATCAGCAGACCGATTCTGGGCGCTGCGTCGCATCGGTGGCAGGACTCCTGAGTATATTTCAGAACTGAACCATTTCAACCGAGCTGAAGCATTCTGCCTTCCTAGTGGTACCAGTCCAATTTAGGAGAGCCAAGCAGACTATCAGTCATCATGGCGAACCTTGGCTACTGGCTGCTGGCCCTCTTTGTGACTATGTGGACTGATGTCGGCCTCTGCAAAAAGCGGCCAAAGCCTGGAGGGTGGAACACCGGTGGAAGCCGGTATCCCGGGCAGGGAAGCCCTGGAGGCAACCGTTACCCACCTCAGGGTGGCACCTGGGGGCAGCCCCACGGTGGTGGCTGGGGACAACCCCATGGGGGCAGCTGGGGACAACCTCATGGTGGTAGTTGGGGTCAGCCCCATGGCGGTGGATGGGGCCAAGGAGGGGGTACCCATAATCAGTGGAACAAGCCCAGCAAACCAAAAACCAACCTCAAGCATGTGGCAGGGGCTGCGGCAGCTGGGGCAGTAGTGGGGGGCCTTGGTGGCTACATGCTGGGGAGCGCCATGAGCAGGCCCATGATCCATTTTGGCAACGACTGGGAGGACCGCTACTACCGTGAAAACATGTACCGCTACCCTAACCAAGTGTACTACAGGCCAGTGGATCAGTACAGCAACCAGAACAACTTCGTGCACGACTGCGTCAATATCACCATCAAGCAGCACACGGTCACCACCACCACCAAGGGGGAGAACTTCACCGAGACCGATGTGAAGATGATGGAGCGCGTGGTGGAGCAGATGTGCGTCACCCAGTACCAGAAGGAGTCCCAGGCCTATTACGACGGGAGAAGATCCAGCAGCACCGTGCTTTTCTCCTCCCCTCCTGTCATCCTCCTCATCTCCTTCCTCATCTTCCTGATCGTGGGATGAGGGAGGCCTTCCTGCTTGTTCCTTCGCATTCTCGTGGTCTAGGCTGGGGGAGGGGTTATCCACCTGTAGCTCTTTCAATTGAGGTGGTTCTCATTCTTGCTTCTCTGTGTCCCCCATAGGCTAATACCCCTGGCACTGATGGGCCCTGGGAAATGTACAGTAGACCAGTTGCTCTTTGCTTCAGGTCCCTTTGATGGAGTCTGTCATCAGCCAGTGCTAACACCGGGCCAATAAGAATATAACACCAAATAACTGCTGGCTAGTTGGGGCTTTGTTTTGGTCTAGTGAATAAATACTGGTGTATCCCCTGACTTGTACCCAGAGTACAAGGTGACAGTGACACATGTAACTTAGCATAGGCAAAGGGTTCTACAACCAAAGAAGCCACTGTTTGGGGATGGCGCCCTGGAAAACAGCCTCCCACCTGGGATAGCTAGAGCATCCACACGTGGAATTCTTTCTTTACTAACAAACGATAGCTGATTGAAGGCAACAGGAAAAAAAAAATCAAATTGTCCTACTGACGTTGAAAGCAAACCTTTGTTCATTCCCAGGGCACTAGAATGATCTTTAGCCTTGCTTGGATTGAACTAGGAGATCTTGACTCTGAGGAGAGCCAGCCCTGTAAAAAGCTTGGTCCTCCTGTGACGGGAGGGATGGTTAAGGTACAAAGGCTAGAAACTTGAGTTTCTTCATTTCTGTCTCACAATTATCAAAAGCTAGAATTAGCTTCTGCCCTATGTTTCTGTACTTCTATTTGAACTGGATAACAGAGAGACAATCTAAACATTCTCTTAGGCTGCAGATAAGAGAAGTAGGCTCCATTCCAAAGTGGGAAAGAAATTCTGCTAGCATTGTTTAAATCAGGCAAAATTTGTTCCTGAAGTTGCTTTTTACCCCAGCAGACATAAACTGCGATAGCTTCAGCTTGCACTGTGGATTTTCTGTATAGAATATATAAAACATAACTTCAAGCTTATGTCTTCTTTTTAAAACATCTGAAGTATGGGACGCCCTGGCCGTTCCATCCAGTACTAAATGCTTACCGTGTGACCCTTGGGCTTTCAGCGTGCACTCAGTTCCGTAGGATTCCAAAGCAGACCCCTAGCTGGTCTTTGAATCTGCATGTACTTCACGTTTTCTATATTTGTAACTTTGCATGTATTTTGTTTTGTCATATAAAAAGTTTATAAATGTTTGCTATCAGACTGACATTAAATAGAAGCTATGATGAACACCTGG

*Scg3* | NM_011170.3 | *Mus musculus*

GGAGCATGCGCCTCCTCCTCATTTCCTCTCCTGGAGCTAGTGGAGTAAAGCTACGCCCAGGCCCGCGTCCGCTGGCGGCGCAGGAACTTCAGCACCCGCGGGGCGGACAGCGCCTACCGCACCTGCTCACCTGCTCTGGGCGCCAGAAGAGCCTGCATCCTCCTTCCAGCCCGGAGCAACTGCGCCGGAGGCGCCCAGACCCTCTCCCTTCCCGCACCCAGGCTCCTGTCCCTTCCAGCTTCTTAACTCCCCTTCTCATTCATAACAAAAGCTACAGCTCAGGGGCCCAGCGCCAAGCTCTTTCCAGCAAAGCACAGAAGAGCAAGAAAGAATGGGGTTCCTTTGGACCGGCTCTTGGATACTGGTGTTGGTGCTCAACAGCGGCCCAATTCAAGCTTTCCCCAAACCCGAAGGCAGCCAAGACAAATCCCTGCATAATAGAGAATTAAGTGCAGAAAGACCTTTGAATGAACAGATCGCTGAGGCAGAGGCAGACAAGATTAAAAAGGCATTCCCTTCAGAAAGCAAGCCGAGTGAAAGCAATTATTCTTCTGTCGATAACTTGAATCTGCTGAGGGCAATAACAGAAAAGGAAACCGTTGAGAAAGAGAGACAATCCATAAGAAGCCCCCCGTTTGATAACCAACTGAACGTGGAAGACGCTGATTCAACCAAAAATCGGAAACTGATCGATGAGTACGATTCCACCAAGAGTGGACTGGACCACAAGTTTCAAGATGACCCAGACGGCCTTCATCAACTGGATGGAACTCCTTTAACTGCTGAAGACATCGTCCATAAGATTGCCACCAGGATTTATGAGGAGAACGACAGAGGAGTGTTTGACAAAATTGTTTCTAAACTGCTGAATCTTGGCCTGATCACTGAAAGCCAGGCACATACTCTGGAAGATGAAGTAGCAGAAGCTTTACAAAAACTGATTTCAAAAGAGGCCAACAATTATGAGGAGACCCTGGATAAACCCACAAGCAGGACCGAGAATCAGGATGGGAAAATACCAGAGAAAGTGACTCCGGTGGCAGCAGTCCAAGATGGCTTCACTAACCGTGAAAACGATGAGACGGTGTCTAACACCTTGACCTTGTCCAATGGCTTGGAAAGGAGAACTAACCCCCACAGGGAAGACGACTTTGAGGAACTCCAGTATTTCCCCAACTTCTATGCACTACTGACAAGCATCGACTCAGAAAAAGAAGCAAAAGAGAAAGAAACCCTGATCACCATCATGAAGACATTGATTGACTTCGTGAAAATGATGGTGAAATACGGTACGATATCTCCAGAGGAAGGCGTGTCCTACCTTGAAAACTTGGATGAAACAATTGCTCTGCAGACCAAGAACAAGCTAGAAAAAAATACTACTGATAGCAAAACTCCACCAGAGAAGAGTCAGGAAGAAACAGACAGTACCAAGGAAGAAGCCGCCAAGATGGAAAAGGAATACGGAAGCCTAAAAGACTCTACAAAAGATGATAACTCCAACCTAGGAGGAAAGACAGATGAAGCCACAGGGAAGACAGAAGCCTACTTGGAAGCCATTAGAAAAAACATCGAATGGCTGAAGAAACATAACAAGAAGGGCAACAAAGAAGATTACGACCTTTCAAAGATGAGGGACTTTATCAACCAACAAGCTGACGCTTATGTGGAGAAGGGCATCCTCGACAAGGAAGAAGCCAACGCCATCAAACGCATCTACAGCAGCCTGTGAAAATGGCGGGCAGCTTGAGCCTTCCTGTTGTTCCAGCAAAAACAATATAGCTTACAAACTAATTCGGCGGTTAAAGGGTTACCAGCCCAGAAGTATTAGGATGTGCTGAATTTATAGTAGTTAATCCCTTAGAAATGAGTAAAATAGAGCTCTCTTGCCATAAATACCTTATGAAAAGCAAAGCTGTAGAGAAGCCGAGGTTTTTCTATATAGAATCCTTATTTCCTCTTGAATTTACATTTTGTAATCAGAGATGTGCTGCTCTGGAAAAGACTCTAATGGGTTGAACATAAGTCTGAACCTACTCCCCACTGTCCTCAGCCCCCTGAAGCTCTGAGAGGCCCTGTCTCGGCATGCTAGACACCTGAGCACCTCACTGGATGTTTGTCATAGGATGTCGTTTCCACTAGTCGATCTCTGTTGGGCACGGAAATAAACCCACGTCTCTTCATATCCTTGA

*CLN3 362* | AAS52304 | *Ashbya gossypii*

GGAGAGTCTGCATACCAAAGATCAGCCGCTTGCATTGAAAGGGACGAACGGTGGCTCATGTCTTTGCCATCCTTTCTTCTCCAATCCCCGAAGCACTTCAGACACACACGTAACCTTGCATGAAATATAGTATTTATTGTCATCGCCGGCTGGACTGTTACTATCTCAAGCCCAAAGACCATTGCATATCACACACAAATTCGTCACAAACACTTAAAGATTGTCCTGCGGTCAGGCTTCTCTGCTACACCGGGCCTCTGTCAGTATGTCTGTTTGGGAACGTAAGTGTTCGCATCACCGGCTAATAAAAGTGGACGTGTTTGCGTAACAAACTGACTGCTCGGAGAACCGCATTTTAACCCTTACGCTTCTGATTGATCTCGTGTATCACACCCGGTAATCATGTACTTGTTTCCAGTCCCGACCCTTAGTTACCGTGGCGATTCGCAAATTGATTGATGATTCTTGCCCTAGTTTCCCGCCATCCTTGTGCTTCCCCTCTTCGACGCTCTTTATAGGCAGCGCCAAATTTTATCGGTAGCGCGCTATGTTCATTGTTATGTCACCCACATATCTTCTTCTTGGGCTTGACACTATTTGGCTGACGTCCAAGTTCGCAGATCTAATGCCCCGACTATCGAGCTTCCAAAGCCTCCGCCGCCACGAGCAAAAGAAACCGCATTTCACGACATCAGAGCCGACAATCCCCAACACCCTTCACTGGTCAGTATGTACTACGGCGACTCTCTATTCATAGGCGGAGATCAACCGGCAAACAAAGATAGGCAGTCTCCAGTTCTGGCCCATCTCTATGAGCAGTTTAAGGTCTCGAGCACCGAATCTTTGCAAGATATTCCCGTATGACCCAGACCCGAAATACAATACCAAGTTCAGCGCCGTTCACTTCGCAAGCGTAAGAGTTCTAAAGACCGTACGCAGGCTCGCTGTGCTCTCCTAGGTCATGGTTTGCTGACGATGTACAACTGACAAAAATCCCATTCCCATCCGCACCCAGGTTCTAGAGGTAGTCGCCAACCCGTCCAACGCAAACTCCAGATCTCCACCCAATTGCCTTTCAGGTGGTGCCTCGATCCTTGCCGTCTAAATTATAGCCAGCCTGTGCCCATTTAACCGACGGTTAAGTAATGAGAAAAAGCGTTCCTGGCGAGATGCAGGCACGGGGGTGGGAATCGAATGGCCAGGCGCCGCAGTGCTCCGTGCTACCTGCCTCTCCCCCGATATCAGCAGTGACGGCGACTCTAGCTCGGGCTCTGTTCCACCATTTACTCTGGACCGGCCCGCCCCGCACAAGACGACACTCAAGACAGCTGTGGCCCCCCTAACATCTACTCCGAACGACTCACCGCGCACTCATCTCACGTATCGTTCGATGCCCCAGACGCCACCCACTATTATGTCCGTTGCAGCTCAGGCGCGTGCACAATGCCTCCCAGACCTACACTCCACCACGACTAAGAACCCCCTTCACTTGCTGCATTCTGAAGAATCTAAGGACTCCGCCACGGGTATCGACCATGAGCTATGCGACATTGTTCAGGACTCGGTCAAGAAACTACGCTAGCATAC

*CLN3 657* | AAS52304	| *Ashbya gossypii*

GGAGAGTCTGCATACCAAAGATCAGCCGCTTGCATCACATGAGACCCTGCGGCACTCTCTTATTTTGCGTAGTTTCTTGGCCAAACTTAGGTAACCCACACTAAGAGGACACTGCCTGCATAAATACTGAAATTCTCTGTTCTCATCATCCGAGCAGTTAATAACTCCTAACGGACCCCATTTGCATCTTATTCGCACATCTGCGAAAGACTCTAGGAAACACCCACGCTCTAAAGCATCAATACTCCTATTGTCCGTAAATGGTACATCCGTGTGGGAAAGTCACGCGCTCCATCGCTGGTTCCAGACCAGAACGATGGAAGCGCATCAATATGCTTGCCAGCATGCCAGTGTTGCACTCCGCAAGCATCTCAGTGATCTGGAGAACCAGAAGCGGGCGTCCGGGACTGGCTTCCCGTACCTGCCGCAACTAGGTATCGGGATGTTCAACTTGAGTTTGCGCCACTTCATGTCCTTCCAAACTCGCTTGCGACTGTCCTAATCCACTCGCCTATCATGGCAAGACATCATGGGGCTTTTCAGCTCCAAGATGGTTTTTACTTCTACCCCCTTTCACCTACATACGCTTACGGCGGTAAGCCTGTAGTGATAGTTCTTACATAAAAACACCCGCACCCCGAGCCCGCAGCGCAATTGCTGCGACAACCGCTCACATGGGCGGTACCATGACCCGGAGCTGACCAACTTCTAGACGCTTCCCTGACCTGCGTATTCAGCTCCGAGTCATGTTTGCGCGCTTCATAACCTACAGATAACCGAGACCCCCAAGCCGTGCTCCATGCTCCTTAATCGAAACAGTTGTGAGCATGGAGCAGGCATAATTGGGTCAGTATGCAGCTCTTACCCAGGAGTTTACTATCATAACTGCTCCAACGCCATTCATATGGCGAGCGTTACCCCTATAACAATCGCATCCCGATATGCTGACCCCAAAGGGTCAGCATGCTGCAGGCCGTGTTTAACAGATGGAAATCTCATGGCGGTCTGCAGCCCCCATCTTTGCCTACAGGCCTGCGATGTCAAATTCCGGGCCTGTCTTTCCCTCTAGTCGGGATCCTGGAGCAGCGTGCGAATCTAAAGCACGACTATGTCTCCTCGGTAACCTTAATACCGCCAGTTAGCCGATGAGATCAATCTGTAGTATGGTTATGCAAGCGCTTCCGTGGGCTCGCCAAGTGCGGGTCTAGCTCCGGCCACTGCGGCCAGCGGCTCTCCCGCTGTCCGCATGGCAGGCAGCTAGACCCCGGCCTATGTCCCGACATCCGCTCTGGTGACCCTTACGCGGCATATGCCGACGATCAAGGCTTTTATGCCGCTCACTCCAACTACTTCTCGCAAGCCAAAGCGCCTAGTGCTCTGGCTTAGTGAAAAGTACCAGACGAGGCCCGCGCCTGTGGAGGTTACATCATAGGCGCGCGCAAAAGGCACTACCCATCAACATTCACCGCCTCTCCAGGAGATCGCGCAGTTGCTGCATCAGTAGCAACATCAGCGCGACCCCATGGCCGTGGACCTCCAGTTCAATGCCCGGGAACCACTCCTGGTCAAGAAACTACGCTAGCATAC

**Supplementary Table S4: Atp5b_DNA oligos sequence for smFISH.**

| Table S4_Atp5b_DNA oligos sequence for smFISH | | |
| --- | --- | --- |
| NAME_start | **Sequence (5'->3')** | **position of the first nucleotide** |
| Atp5b_p39 | GCCCCACAAGACTCAACA | 39 |
| Atp5b_p160 | GCATAGTCTCTGGCAGGA | 160 |
| Atp5b_p275 | GGCATTTAGGATGGGTGG | 275 |
| Atp5b_p354 | CAATAGTTCTGACCGTGCTC | 354 |
| Atp5b_p375 | AGCCTTCAGTGCCATCCA | 375 |
| Atp5b_p399 | CCAGTACTTTCTGGCCTC | 399 |
| Atp5b_p424 | GGAATTTTGATTGGTGCCCC | 424 |
| Atp5b_p490 | GGACCTCTCTCATCAATAGG | 490 |
| Atp5b_p545 | CTCTATGAACTCAGGAGCC | 545 |
| Atp5b_p577 | CCAGTCACCAGAATCTCC | 577 |
| Atp5b_p625 | ATTTTCCCACCCTTGGCG | 625 |
| Atp5b_p675 | GCTCCATGATCAGTACTGTC | 675 |
| Atp5b_p756 | AATCATTGCCCTCACGGG | 756 |
| Atp5b_p831 | GTCCATATACCAACGCTACC | 831 |
| Atp5b_p880 | AAACCAGTCAGAGCTACCC | 880 |
| Atp5b_p960 | CCTGGGTAAAGCGGAAGA | 960 |
| Atp5b_p988 | CCCAATAAGGCAGACACC | 988 |
| Atp5b_p1011 | AGCCTACAGCAGAAGGGA | 1011 |
| Atp5b_p1066 | GTGGTGGTGATCCTTTCC | 1066 |
| Atp5b_p1111 | GCAGGCACATAGATAGCC | 1111 |
| Atp5b_p1162 | GCATCCAAATGGGCAAAGG | 1162 |
| Atp5b_p1210 | GCTGGATAGATGCCCAAC | 1210 |
| Atp5b_p1326 | CCTGGAGAGATTTGTAGTCC | 1326 |
| Atp5b_p1453 | ATGTGACCCGTGAAGACC | 1453 |
| Atp5b_p1537 | GCTTGTTCTGGGAGATGG | 1537 |
| Atp5b_p1561 | TCAATGGGTCCCACCATG | 1561 |
| Atp5b_p1648 | GAGAGATCAGTTGCAGTGC | 1648 |
| Atp5b_p1678 | GGAAGGAAACCTGAGCTC | 1678 |
| Atp5b_p1701 | CAAGGCTCTTGTGTGGCC | 1701 |
| Atp5b_p1831 | CTAAATGTGGCCCACCAC | 1831 |

**Supplementary Table S5: Antibody used in this study.**

| Table S5_Antibodies in use | | | | | |
| --- | --- | --- | --- | --- | --- |
| Name | **Type** | **Conc. in use in this study** | **Application in this study** | **Source** | **Identifier** |
| anti-vGLUT1 | polyclonal rabbit | 1/500 | ICC in WT neurons | Takamori et al., 2001 | Shigeo3 |
| anti-MAP2 | polyclonal chicken | 1/1000 | ICC in WT neurons | kind gift from Suzanne Wegmann's lab | n/a |
| anti-EIF4E | monoclonal rabbit | 1/250 | ICC in HEK293 | Abcam | ab33766 |
| anti-rabbit STAR 488 | goat anti-rabbit | 1/500 | ICC in HEK293 | Aberior | ab464 |
| anti-chicken AF488 | anti-chicken | 1/500 | ICC in WT neurons | kind gift from Suzanne Wegmann's lab | n/a |
| anti-rabbit AF568 | goat anti-rabbit | 1/500 | ICC in WT neurons | Alexa Fluor | A-11001 |
| anti-betaActin | monoclonal mouse | 1/2000 | WB | Sigma Aldrich | A5441-0.2ML |
| anti-mCherry | monoclonal mouse | 1/2000 | WB | Abcam | Ab125096 |
| anti-mouse HRP | anti-mouse IgG | 1/10000 | WB | Sigma Aldrich | A4416-1ML |
| anti-PSD95 STAR 635P | sdAb | 1/500 | ICC in WT neurons | NanoTag Biotech. GmbH | AB_307  6102 |
